# A General Self-Sacrifice Template Strategy to 3D Heteroatom-Doped Macroporous Carbon for High-Performance Potassium-Ion Hybrid Capacitors

**DOI:** 10.1007/s40820-021-00659-7

**Published:** 2021-05-29

**Authors:** Junwei Li, Xiang Hu, Guobao Zhong, Yangjie Liu, Yaxin Ji, Junxiang Chen, Zhenhai Wen

**Affiliations:** CAS Key Laboratory of Design and Assembly of Functional Nanostructures, and Fujian Provincial Key Laboratory of Nanomaterials, Fujian Institute of Research On the Structure of Matter, Chinese Academy of Sciences, Fuzhou, Fujian 350002 People’s Republic of China

**Keywords:** Potassium-ion hybrid capacitors, Self-sacrifice template, Se/N co-doped, 3D macroporous, Long service life

## Abstract

Potassium-ion hybrid capacitors (PIHCs) tactfully combining capacitor-type cathode with battery-type anode have recently attracted increasing attentions due to their advantages of decent energy density, high power density, and low cost; the mismatches of capacity and kinetics between capacitor-type cathode and battery-type anode in PIHCs yet hinder their overall performance output. Herein, based on prediction of density functional theory calculations, we find Se/N co-doped porous carbon is a promising candidate for K^+^ storage and thus develop a simple and universal self-sacrifice template method to fabricate Se and N co-doped three-dimensional (3D) macroporous carbon (Se/N-3DMpC), which features favorable properties of connective hierarchical pores, expanded interlayer structure, and rich activity site for boosting pseudocapacitive activity and kinetics toward K^+^ storage anode and enhancing capacitance performance for the reversible anion adsorption/desorption cathode. As expected, the as-assembled PIHCs full cell with a working voltage as high as 4.0 V delivers a high energy density of 186 Wh kg^−1^ and a power output of 8100 W kg^−1^ as well as excellent long service life. The proof-of-concept PIHCs with excellent performance open a new avenue for the development and application of high-performance hybrid capacitors.

**Supplementary Information:**

The online version contains supplementary material available at 10.1007/s40820-021-00659-7.

## Highlights

- A newly versatile self-sacrifice method to fabricate selenium and nitrogen co-doped 3D macroporous carbon was developed.
- The K^+^ storage mechanism of Se and N active sites was deeply studied by systematic electrochemical characterization combined with theoretical calculations.
- The assembled K-ion hybrid capacitor possesses high energy/power densities and excellent cycling stability for practical applications.

**Supplementary Information:**

The online version contains supplementary material available at 10.1007/s40820-021-00659-7.

## Graphical abstract

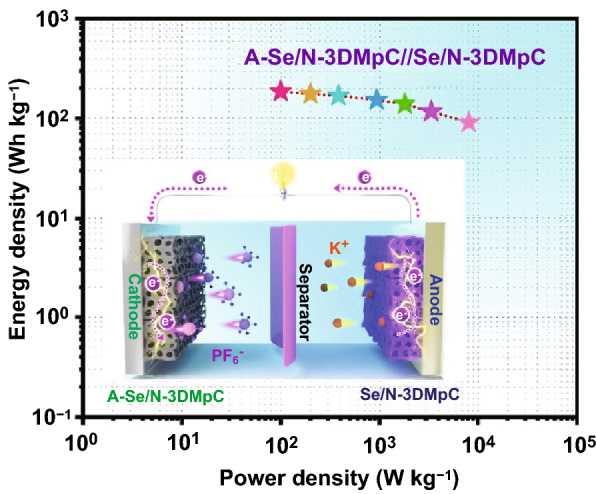

## Introduction

Potassium-ion hybrid capacitor (PIHC), as an emerging new energy storage device, has recently been proposed to bridge the gap between high power density of potassium-ion batteries (PIBs) and high energy density of supercapacitor (SC) by integrating complementary merits of the two systems [1–4]. Unfortunately, the potassium-ion storage anode usually suffers from the issues of dramatic volume variation and fast capacity fading because of the intrinsic large-sized K^+^, which causes slow redox reaction kinetics behavior upon K^+^ intercalation/deintercalation process [5–8]. In addition, the activated carbon, as the typical capacitor-type cathode of PIHCs, shows fast charge/discharge rate, while it tends to deliver limited capacitance [6, 9]. Therefore, the development of desired electrode materials to overcome the imbalance of kinetics and capacity between the anode and cathode in PIHCs is urgently needed, yet remains grand challenge so far [10–12].

A diversity of materials, such as metal alloys [13, 14], selenides [5], oxides [15, 16], MXene [17, 18], and carbon-based material [19] have been studied as battery-type anodes. Among them, carbon-based materials [10, 20–23] with the intercalation mechanism are considered as one of the most promising anode candidates for large-scale practical applications thanks to the attractive merits of low cost and high electrical conductivity. Therefore, various carbon-based materials, including graphene [24], hard carbon microspheres [25], soft carbon [26], hard-soft composite carbon [27], nanocrystalline graphite [28], and carbon nanofibers [29], have been extensively studied as anode of PIHCs. Recently, the theoretical calculations show that the N-doped, P/N co-doped, and S/N co-doped carbon-based materials can effectively adjust the localized electrons near the heteroatoms, increase electron negativity of defective sites, and improve the electrical conductivity of carbon, thereby promoting the potassium storage performance [3, 4, 30]. Nevertheless, there remains space to further improve the overall performances of PIHCs for stepping forward its practical application. On the one hand, relatively narrow interlayer space in the traditional battery-type carbon anode likely led to low capacity and unsatisfactory rate. On the other hand, activated carbon (AC) is commonly employed as cathode material for PIHCs due to its large specific surface area (SSA) [9], and its rather low level of activity sites limited the specific capacity of cathode, presenting another critical factor encumbering the overall performance of PIHCs.

Herein, density functional theory (DFT) calculations are performed to screen selenium (Se) and nitrogen (N) co-doped carbon as one promising material for K^+^ storage. To this end, we develop a newly versatile self-sacrifice method to fabricate Se and N co-doped three-dimensional (3D) macroporous carbon (Se/N-3DMpC) by using SeO_2_ powder as the Se source and self-sacrifice template. The desired features in Se/N-3DMpC, including connective hierarchical pores, expanded interlayer structure, good conductivity, and rich activity site, are greatly beneficial to facilitate K^+^ reversible insertion/extraction and to increase the redox sites for capacitor-type cathode. As expected, the resultant Se/N-3DMpC exhibits superior K^+^ storage capability and impressively high capacitance and thus shows promise to be applied as both anode and cathode of PIHCs by manifesting high energy/power density and long cycling stability.

## Experimental Section

### Synthesis of Se/N-3DMpC

100 mg chitosan, 200 mg urea, and 200 mg SeO_2_ were first ground to mix well. The mixture was then transferred into an autoclave and heated to 200 °C for 3 h to gain the precursor. The precursor was then loaded in the porcelain boat and placed at the middle position of a quartz tube. The Se/N-3DMpC was ultimately obtained through high-temperature treatment at 800 °C for 2 h under Ar atmosphere with a heating rate of 5 °C min^−1^.

### Synthesis of Se-3DMpC, N-pC, and Pure-C

The procedure for the preparation of Se-3DMpC is similar to Se/N-3DMpC except that the urea was removed. The procedure for the preparation of N-doped porous carbon (N-pC) is similar to Se/N-3DMpC except that the SeO_2_ was removed. The procedure for the preparation of pure carbon (pure-C) is similar to Se/N-3DMpC except that the SeO_2_ and urea were removed.

### Synthesis of A-Se/N-3DMpC

As-prepared Se/N-3DMpC and KOH were thoroughly mixed with the mass ratio of 1:4. The mixture was heated at 800 °C for 1 h under Ar atmosphere with a heating rate of 5 °C min^−1^. The activated sample was washed with 1.2 M HCl aqueous solution to remove additional KOH and then washed with deionized water (DI) for several times. Finally, the A-Se/N-3DMpC was freeze-dried for more than 72 h at − 40 °C with a freeze-dryer under 0.05 mbar of pressure.

### Synthesis of PVP-, Starch-, and Glucose-Based Se-Doped 3DMpC and Se/N Co-Doped 3DMpC

The procedure for the preparation of PVP-, starch-, and glucose-based Se-doped 3DMpC is similar to Se-3DMpC except that the chitosan was replaced by PVP, starch, and glucose. The procedure for the preparation of PVP-, starch-, and glucose-based Se/N co-doped 3DMpC is similar to Se/N-3DMpC except that the chitosan was replaced by PVP, starch, and glucose.

### Material Characterizations

The morphologies of synthesized samples were inspected by field emission scanning electron microscopy (FESEM, Hitachi SU-8020). Field emission transmission electron microscopy and high-resolution transmission electron microscopy (FETEM, Tecnai F20) were used to observe the detailed structures of as-prepared samples. The X-ray diffraction (XRD, Miniflex 600 powder X-ray diffractometer with Cu Kα radiation at a scan rate of 5° min^−1^) and Raman spectrum (LabRam HR800) and X-ray photoelectron spectroscopy (XPS, ESCALAB 250Xi, Thermo Fisher) were employed to analyze the defects and detailed structures. Nitrogen adsorption–desorption isotherms were analyzed by an intelligent gravimetric sorption analyser (IGA100B). The pore-size distribution was achieved employing density functional theory (DFT) model. The Se/N-3DMpC electrodes were taken out of the half-battery at different stages in an argon-filled glove box and then were washed with diethyl carbonate before ex situ Raman, XPS, XRD, SEM, and TEM tests.

### Electrochemical Measurements

The Se/N-3DMpC, Se-3DMpC, and N-pC electrodes were prepared by coating a slurry containing active materials, acetylene black, and sodium carboxymethyl cellulose in a weight ratio of 8:1:1 on copper foil. Then, the as-prepared electrode was dried at 100 °C overnight and compressed at 5 MPa pressure. The mass loading of the active materials on each electrode was around 1.0–1.2 mg cm^−2^. All the electrodes were assembled with potassium metal counter electrode and Whatman glass fibers into a 2032 model cells in an Ar-filled glove box (< 0.01 ppm of oxygen and water). 0.8 M KPF_6_ in ethylene carbonate (EC)/diethyl carbonate (1:1, v/v) was employed as the electrolyte. The electrochemical measurements of half-batteries were performed by a LAND CT2001 test system between 0.01 and 3.0 V at room temperature. The cyclic voltammetry (CV) curves (0.1 mV s^−1^) were obtained by a CHI660E electrochemical workstation. Electrochemical impedance spectroscopy was carried out at a frequency range from 0.01 Hz to 100 kHz.

A coin-type PIHC full cell was constructed employing the pre-potassiation Se/N-3DMpC anode and A-Se/N-3DMpC cathode. The mass ratio of Se/N-3DMpC (anode) and A-Se/N-3DMpC (cathode) was 1:4. The A-Se/N-3DMpC cathode was constructed by coating a slurry containing A-Se/N-3DMpC (80 wt%), acetylene black (10 wt%), and sodium carboxymethyl cellulose (10 wt%) on aluminum foil. Before the assembling of the PIHCs devices, the Se/N-3DMpC anode was activated in half batteries at 0.1 A g^−1^ for three cycles and ended with a potassiation state of 0.01 V. The energy (E, Wh kg^−1^) and power densities (P, W kg^−1^) of assembled PIHCs were calculated employing the equations as follows:

1 $$P = \Delta V \times i$$

2 $$E = P \times t/3600$$

3 $$\Delta V = (V_{\max } + V_{\min } )/2$$

where *t* (s) is the discharge time, *i* (A g^−1^) is the charge/discharge current, *V*_max_ (V) is the discharge potential excluding the IR drop, and *V*_min_ (V) is the potential at the end of discharge voltages.

### Calculation Method

The density functional theory (DFT) calculations were performed with periodic super-cells under the generalized gradient approximation (GGA) using the Perdew–Burke–Ernzerhof (PBE) function for exchange–correlation. The interaction of ion–electron is described by projected augmented wave (PAW) pseudopotentials. The Kohn–Sham orbitals were expanded in a plane-wave basis set with a kinetic energy cutoff of 30 Ry and the charge density cutoff of 300 Ry. The Fermi surface effects have been treated by the smearing technique of Methfessel and Paxton, using a smearing parameter of 0.02 Ry. The Brillouin zones were sampled with a k-point mesh of 1 × 1 × 1. The calculation model of the pure, Se-, N-, and dual (Se, N)-doped carbon was constructed with 5 × 5 lateral periodicity shown in Fig. S1. The vacuum layer was ∼ 15 Å to remove the interaction of adjacent atomic slabs in the *z* direction. The nudged elastic band (NEB) method was used to evaluate the transition state and activation energy for one K-ion migrate from one hollow site to the nearest hollow site in the carbon. The graphitic interlayer spacing of the pure-C, N-pC, Se-3DMpC, and Se/N-3DMpC was set at 3.4, 3.6, 3.88, and 4.04, respectively, according to the results of XRD (Fig. 2g). By adding the adsorbate in each model and then the geometry structures were optimized again (shown in Fig. S2). Subsequently, the structure of potassium ions adsorbed on different carbon models can be obtained, and the corresponding Δ*E*_a_ can be calculated by Eq. 4:

4 $$\Delta E_{{\text{a}}} = E_{{{\text{total}}}} - E_{{{\text{adsorbate}}}} - E_{{{\text{structure}}}}$$

where *E*_total_ is the total energy of compound, *E*_adsorbate_ is the energy of adsorbate, and *E*_structure_ is the energy of corresponding structure. Additionally, the energy of one potassium atom is calculated by the energy of per potassium atom in potassium metal phase. Our DFT calculations are implemented by the PW and NEB modules contained in the Quantum ESPRESSO distribution [31].

## Results and Discussion

### DFT Analysis of the Effect of Se and N Dopants on Potassium-Ion Storage

To explore the role of Se and N co-dopants in carbon-based material for PIHCs, the DFT calculations were conducted. The adsorption energy (Δ*E*_a_) of K^+^ for the diversity of geometry configurations is imported as the descriptor for adsorption stability. Therefore, the models of pure carbon, Se-doped, N-doped, and Se/N co-doped carbon were constructed as host materials with optimization (Fig. S1). The Δ*E*_a_ of K^+^ on the activity sites in all configurations was calculated (Fig. S2). The pure carbon shows a Δ*E*_a_ of 0.47 eV (Fig. S2a), suggesting the dopant-free carbon cannot offer an effective adsorption for K^+^. With importing N element, it was found that the K^+^ adsorption on the site neighbor to the edge N species is obviously enhanced (Fig. S2b). Therefore, N doping can effectively improve the adsorption capacity for K^+^ (− 1.60 eV) on carbon materials. Meanwhile, Se doping in the carbon matrix can also enhance the interaction between K^+^ and carbon, affording a Δ*E*_a_ of − 0.04 eV (Fig. S2c). Additionally, the larger radius of Se atom can cause obvious protrusions in the carbon surfaces, increasing the interlayer spacing between the carbon layers and thus fortifying the capacity for potassium-ion storage (Fig. S1c). Furthermore, the Δ*E*_a_ of K^+^ on the Se-doped carbon can be further enhanced with the additional doping of N, resulting in a favorable Δ*E*_a_ of − 1.61 eV for K^+^ adsorption (Fig. S2d). Overall, N/Se co-doping in carbon-based materials can be a promising strategy to promote the K^+^ storage performance.

**Fig. 1:**
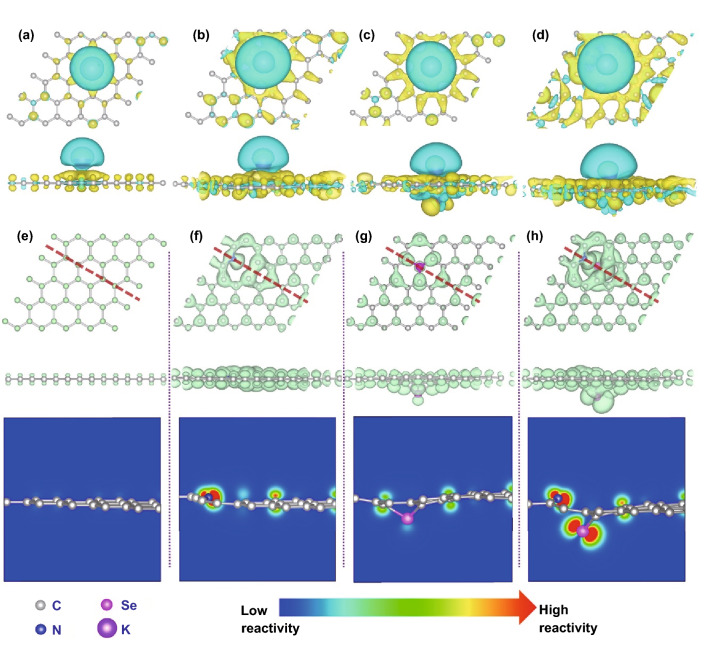
Top and side illustration of the calculated charge density difference of the K^+^ adsorbed on **a** pure carbon, **b** N-doped carbon, **c** Se-doped carbon, and **d** Se/N co-doped carbon. Top and side illustration of the local Fermi softness [S_F_(r)] and the corresponding sectional reactivity heat map for the active sites of the **e** pure carbon, **f** N-doped carbon, **g** Se-doped carbon, and **h** Se/N co-doped carbon, where the red and blue colors indicate the higher and lower reactivity, i.e., how easy it bonds with other atoms. The charge density isosurface is set to 0.004 e Å^−3^ in all models

To further investigate the bonding properties of the adsorbed K^+^ in each model, the charge density difference was calculated by subtracting the charge density of K^+^ and each configuration from the corresponding compound (Fig. 1a–d). Compared with undoped carbon, single heteroatoms (Se or N) carbon shows higher density of transferred charges, confirming that single doping of Se or N can increase the adsorption ability of K^+^ on the carbon surface. Moreover, the highest density of transferred charges between the adsorbed K^+^ and substrate can be observed in the Se/N co-doped carbon, demonstrating that co-doping of Se/N is more conducive to improving the adsorption ability of K^+^ on the carbon surface. To obtain an insightful view of the doping effects, we analyzed the partial density of states (PDOS) of p orbitals of N and Se atoms in the Se-, N-, and Se/N-doped carbon to investigate the electronic structure (Fig. S3). The results show that the introduction of Se atom can effectively improve the PDOS of N atom near the Fermi level and vice versa, finally enhancing the adsorption energy of K^+^ in the Se/N co-doped carbon [32]. Furthermore, the Se/N co-doped carbon shows higher reactivity than others according to the associated Fermi softness analysis [*S*_*F*_(*r*)] (Fig. 1e–h), which is consistent with the results of PDOS analysis [33]. The local *S*_*F*_(*r*) is the weighted sum of the contributions from the frontier orbitals, where the higher spatial proportion of the same *S*_*F*_(*r*) suggests the associated active site can have a stronger bond with the potassium ion. The above results clearly demonstrate that the PDOS near the corresponding Fermi level can be mutually strengthened with the co-doping of Se and N in carbon, resulting in an increased adsorption energy for K^+^, and endowing the Se/N co-doped carbon be a promising electrode material for PIHCs.

### Synthesis and Characterizations of the Se/N-3DMpC

**Fig. 2:**
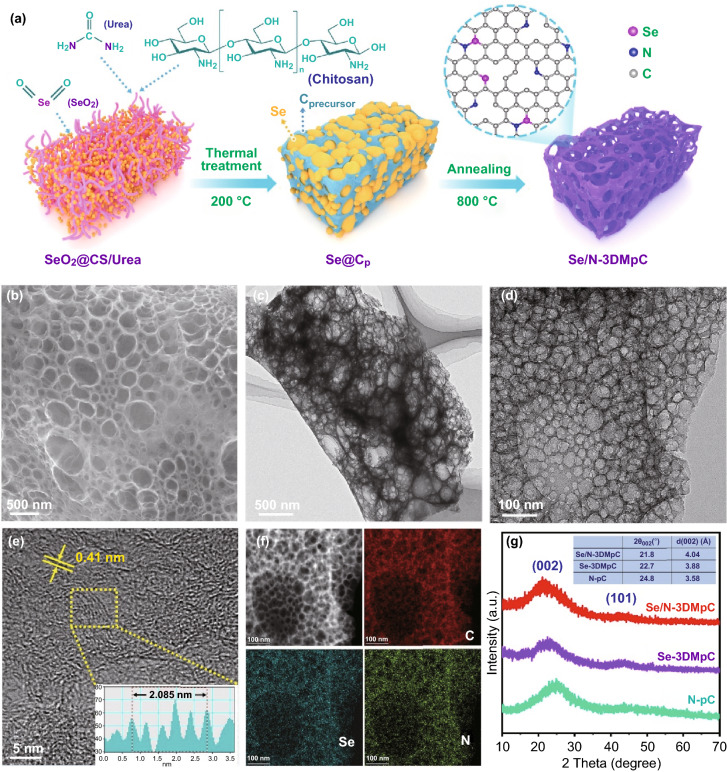
**a** Schematic illustration for preparation of Se/N-3DMpC. **b** FESEM images, **c, d** TEM images, **e** HRTEM image, **f** HAADF-STEM image and EDS elemental mappings of the Se/N-3DMpC, **g** XRD pattern of the Se/N-3DMpC, Se-3DMpC, and N-pC

Steered by the theoretical calculation, we designed a simple one-pot route to synthesize 3D macroporous carbon (Se/N-3DMpC) with connective hierarchical pores, as illustrated in Fig. 2a. First, the mixtures of chitosan (CS), urea, and SeO_2_ were preheated at 200 °C for 3 h, during which the SeO_2_ particles tend to be encapsulated by polymer evolved from CS and urea, and then gradually reduced to Se with increasing the temperature. The subsequent pyrolysis at 800 °C under Ar atmosphere yields the Se/N-3DMpC after evaporating the Se particles and carbonized the polymer. SeO_2_ here is not only used as the template for generating interconnective macroporous structure but also applied as the Se source for doping. For comparison, Se-doped 3D macroporous carbon (Se-3DMpC) without using urea, N-doped microporous carbon (N-pC) without adding selenium source, and pure carbon (pure-C) without urea and selenium source were also synthesized by the similar route to prepare Se/N-3DMpC.

FESEM and TEM were employed to study the process of the formation of the Se/N-3DMpC. The sphere-like Se particles were embedded in carbon precursor matrix before pyrolyzation, as verified by the SEM images, the EDS element mapping, and the XRD of the samples obtained at 200 °C (Fig. S4). The obtained Se/N-3DMpC and Se-3DMpC obviously show macroporous structure thanks to removing Se particles upon high-temperature pyrolysis (Figs. 2b and S5, S6a, b). The nitrogen adsorption–desorption tests indicate both Se/N-3DMpC and Se-3DMpC consist of hierarchical porous structure with considerably high surface area of 711.86 and 652.50 m^2^ g^−1^, respectively (Fig. S7). Notably, the N-pC obtained without using SeO_2_ displays a large amount of stacking thin carbon sheets (Fig. S8), indicating that the gas produced during the high-temperature pyrolysis of urea is beneficial to the formation of thin carbon during the carbonization process. As shown in the TEM images in Figs. 2c–d and S6c–d, both Se/N-3DMpC and Se-3DMpC indeed have a hierarchical interconnective porous structure, which is caused by the removal of Se template and gas generated in the process of pyrolysis. High-resolution TEM (HRTEM) image reveals that the Se/N-3DMpC has an expanded interlayer distance of 0.41 nm (Fig. 2e), which can be conducive to the fast kinetic of insertion/extraction of K^+^. As expected, the interlayer spacing of Se/N-3DMpC is significantly larger than that of Se-3DMpC (0.39 nm) and N-pC (0.36 nm), respectively (Figs. S6e and S8e). The high-angle annular dark-field scanning transmission electron microscopy (HAADF-STEM) image and the corresponding EDS mappings demonstrate homogeneous distribution of Se, N, and C in the porous skeleton of Se/N-3DMpC (Fig. 2f). Interestingly, by replacing different carbon sources (e.g., glucose, starch, and PVP), Se-doped and Se/N co-doped 3D macroporous carbon (3DMpC) can also be obtained (Figs. S9–S12), proving the general applicability of the preparation method.

The enlarged graphite-like interlayer space in Se/N-3DMpC was further proved by powder XRD measurement, two broad peaks of (002) and (100) diffractions indicating the formation of graphite-like carbon for all the samples. The (002) peaks of Se/N-3DMpC, Se-3DMpC, and N-pC are located at around 21.8°, 22.7°, and 24.8°, respectively, corresponding to interlamellar spacing of 4.04, 3.88, and 3.58 Å according to the Bragg’s law [34]. The enlarged interlayer space of Se/N-3DMpC can be attributed to the abundant heteroatoms (Se and N) doped in the carbon layer, which is consistent with the HRTEM results. The Raman spectroscopy was performed to further investigate the microstructure of Se/N-3DMpC, Se-3DMpC, and N-pC. As shown in Fig. S13, two characteristic peaks at 1348 cm^−1^ (D band) and 1580 cm^−1^ (G band) correspond to the breathing vibration of the hybridized carbon rings and stretching vibration of *sp*^2^-type graphitized carbon. The intensity ratio of D band to G band (*I*_D_/*I*_G_) of the Se/N-3DMpC exhibits the highest value (1.10) than those of the Se-3DMpC (1.07) and N-pC (1.04), indicating more structural defects in the Se/N-3DMpC caused by the co-doping of Se and N. XPS further reveals the elemental composition and chemical bonding state of all samples (Fig. S14). The surface elemental composition of Se/N-3DMpC consisted of N (10.48 at%) and Se (2.62 at%) species. The high-resolution Se 3d spectrum of the Se/N-3DMpC and Se-3DMpC displays two fitted peaks of Se 3d_5/2_ (55.7 eV) and Se 3d_3/2_ (56.6 eV) that caused by the spin–orbit coupling (Fig. S15a–b). Moreover, the binding energies of these two peaks are higher than 55.2 eV and 56.1 eV of pure Se, respectively, suggesting that Se atoms were successfully doped into the carbon frame to form Se–C bonds [35]. Meanwhile, a fitting peak suggesting the formation of C–Se/C–N bonds was also observed in the C 1 s spectrum (Fig. S15c). The species of pyridinic (398.2 eV), graphitic (400.7 eV), pyrrolic (399.6 eV), and oxidized (403.3 eV) N are present in the high-resolution N 1 s spectrum of Se/N-3DMpC (Fig. S15d), implying elemental N has also been doped in Se/N-3DMpC. Besides, the species of N are not detected in the survey spectrums of pure-C and Se-3DMpC (Fig. S14), illustrating that the N element of Se/N-3DMpC and N-pC was derived from urea rather than CS. The results of XPS analysis suggest the chemically bonded Se and N have been successfully doped into the Se/N-3DMpC, which contribute to promoting surface capacitive capacity.

### Electrochemical Performance of Se/N-3DMpC Anode

**Fig. 3:**
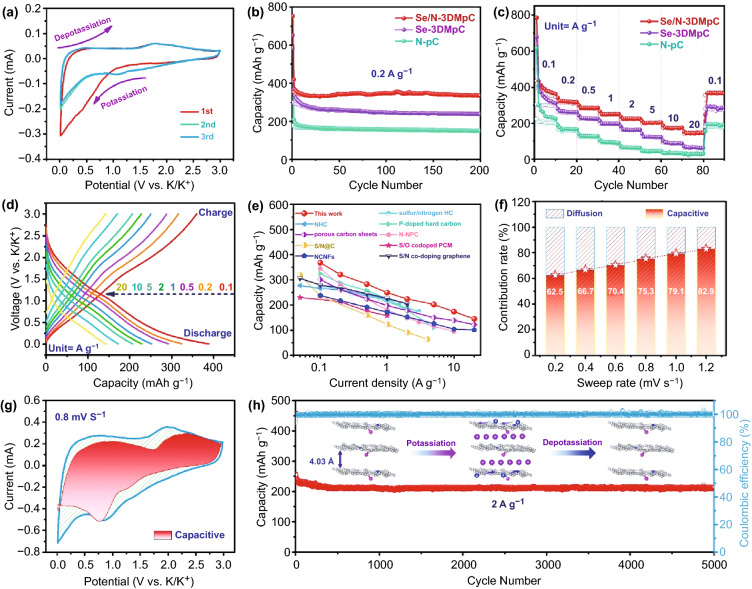
**a** CV curves at a sweep rate of 0.1 mV s^−1^ of the Se/N-3DMpC. **b** Cycling performance of the Se/N-3DMpC, Se-3DMpC, and N-pC at 0.2 A g^−1^. **c** Rate performance of Se/N-3DMpC, Se-3DMpC, and N-pC. **d** Charge–discharge curves at diversity current densities for Se/N-3DMpC. **e** Comparison of rate performance of Se/N-3DMpC with the reported carbon-based materials for PIBs. **f** Contribution of the surface process of the Se/N-3DMpC at different scan rates. **g** Capacitive contribution at 0.8 mV s^−1^ of Se/N-3DMpC electrode. **h** Long-term cycling performance of the Se/N-3DMpC at a current density of 2.0 A g^−1^

The K^+^ storage performance of the as-prepared carbon-based materials was investigated in potassium-ion half-cell with metallic potassium foil as both the counter and reference electrodes. Figure 3a displays the first three cyclic voltammetry (CV) profiles of the Se/N-3DMpC at a scan rate of 0.1 mV s^−1^ between 0.01 and 3.0 V. There is an obvious cathodic peak located at around 0.52 V in the first cathodic scan, corresponding to the irreversible reactions of the decomposition of electrolyte and the formation of a stabilized solid electrolyte interphase (SEI) film [36]. There is a sharp cathodic peak at around 0.01 V, which is ascribed to the formation of K-intercalation compound (KC_8_) by insertion of K^+^ into the interlayer [37]. In the CV curves of Se-3DMpC and N-pC, similar peaks are also observed (Fig. S16a–b). During the anodic process, the peak at around 0.29 V is related to the extraction of K^+^ from the interlayers of Se/N-3DMpC. An anodic peak at around 1.75 V can be observed in the CV profiles of Se/N-3DMpC and Se-3DMpC electrodes, but not in N-pC electrode, which corresponds to the reaction of potassium ions with doped selenium (Figs. 3a and S16a–b) [38]. In addition, the following CV curves of Se/N-3DMpC almost overlap each other, suggesting a good reversibility. Compared with the CV curves of Se/N-3DMpC, Se-3DMpC, and N-pC electrodes, the Se/N-3DMpC electrode presents the largest area, indicating that Se/N-3DMpC has the highest potassium-ion storage capacity among all samples (Fig. S16c–d).

Figure S17a–c presents the galvanostatic charge–discharge (GCD) curves of all species at 0.2 A g^−1^. The voltage plateaus are well consistent with the redox peaks observed in the CV curves. The initial discharging and charging capacities of Se/N-3DMpC are 757 and 411 mAh g^−1^, corresponding to an initial Coulombic efficiency (ICE) of 54.3%. The initial capacity loss of the Se/N-3DMpC electrode might be caused by the consumption of electrolyte and the formation of the SEI layer [39]. Compared with the ICE of Se-3DMpC (45.7%) and N-pC (30.5%), the Se/N-3DMpC shows a considerably improved ICE (Fig. S17a–c), indicating that co-doping of Se and N can significantly increase the first reversible capacity of the carbon-based material. Additionally, the ICE value of the Se/N-3DMpC electrode surpasses most of the previously reported carbon-based materials (Table S1). Furthermore, the Se/N-3DMpC shows a lower voltage drop of 123 mV than Se-3DMpC (305 mV) and N-HPC (436 mV), indicating the conductivity is enhanced by co-doping with Se/N heteroatoms (Fig. S17d).

Figure 3b shows the comparison of the cycling performance of the set of electrodes at 0.2 A g^−1^. After 200 cycles, the Se/N-3DMpC electrode outperforms the other two electrodes by delivering a reversible specific capacity of 335 mAh g^−1^, corresponding to a capacity retention of 95.9%. Although the Se-3DMpC and N-pC electrodes exhibit a similar cyclability, their capacities after 200 cycles are only 238 and 149 mAh g^−1^, respectively. Moreover, the Coulombic efficiency of Se/N-3DMpC electrode increased to 100% after the first few cycles (Fig. S18). Figure 3c displays the rate performance of the all samples from low (0.1 A g^−1^) to ultrahigh (20.0 A g^−1^) current densities. The reversible capacities of Se/N-3DMpC are 368, 321, 283, 249, 224, 202, 174, and 145 mAh g^−1^ at 0.1, 0.2, 0.5, 1, 2, 5, 10, and 20 A g^−1^, respectively, outperforming the Se-3DMpC and N-pC counterparts, thanks to the multi-effects of 3D hierarchical porous architecture, expansion of interlayer space, and abundant heteroatoms (Se and N) doping (Fig. S19). In addition, the charge/discharge profiles of the Se/N-3DMpC electrodes show decreased polarization relative to those of Se-3DMpC and N-pC (Figs. 3d and S20). Such 3D porous structural feature can reduce the volume change of carbon during K^+^ insertion/extraction and enhance the surface capacitance-controlled behavior under high current density. In this context, the rate performance of Se/N-3DMpC electrode is even superior to the previous carbon-based anodes (Fig. 3e) [10, 30, 40–46]. To verify the excellent rate performance of the Se/N-3DMpC anode, CV measurements with diversity sweep rates between 0.2 and 1.2 mV s^−1^ were performed. The contribution of surface capacitance gradually ascends from 62.5 to 82.9% in Se/N-3DMpC electrode with increasing the scan rate, which is greater than those of Se-3DMpC and N-pC at the same sweep rate (Figs. 3f–g and S21–S23). Such high capacitive energy storage behavior of the Se/N-3DMpC electrode is highly correlated with the characteristics of its hierarchical porous structure, which is consistent with previous work [47, 48].

Notably, the Se/N-3DMpC electrode maintains a high reversible capacity of 214 mAh g^−1^ at a high current density of 2 A g^−1^ even after 5,000 cycles, indicating that the Se/N-3DMpC electrode has excellent durability (Fig. 3 h). On the contrary, the performance of the Se-3DMpC and N-pC electrodes gradually decayed and only maintained a reversible capacity of 97 and 3 mAh g^−1^ after 5,000 cycles, which further demonstrated the excellent cycle stability of the Se/N-3DMpC electrode (Fig. S24). The cycle stability of Se/N-3DMpC is better than most materials reported in the previous literature (Table S2). More importantly, the morphology of the Se/N-3DMpC electrode did not change significantly after long-term operation, indicating the superior stable structure (Fig. S25). Additionally, electrochemical impedance spectroscopy (EIS) was conducted to further analyze the electrochemical behaviors (Fig. S26). The results show that Se/N-3DMpC possesses the smallest diameter of semicircle than the others, indicating that charge-transfer resistance (*R*_ct_) of Se/N-3DMpC is lower than that of Se-3DMpC and N-pC. Simultaneously, the slope of straight line of Se/N-3DMpC electrode is the smallest among all electrodes. The results prove the additional Se/N dopants in the carbon-based materials can contribute to fast K^+^ transport kinetics.

### Reversibility and Kinetic Analysis of Se/N-3DMpC Anode

**Fig. 4:**
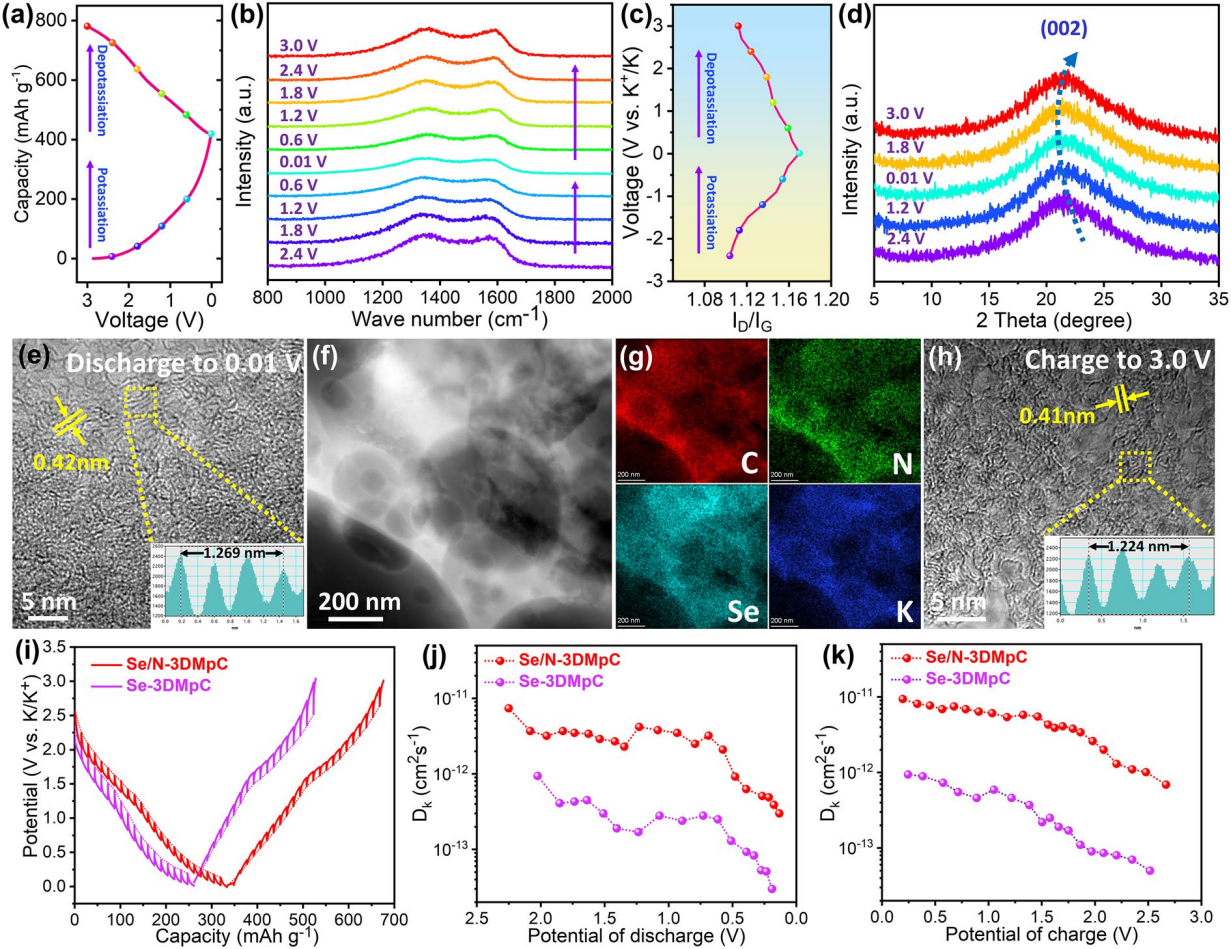
**a** Second discharge/charge cycle and the corresponding voltage position, **b** ex situ Raman spectra at 0.2 A g^−1^, **c** corresponding I_D_/I_G_ ratio, **d** ex situ XRD patterns, **e** HRTEM at fully discharged, **f** HAADF-STEM image, **g** elemental mappings, and **h** HRTEM at fully charged of Se/N-3DMpC. **i** Galvanostatic intermittent titration technique (GITT) curves of Se/N-3DMpC and Se-3DMpC at the 200th cycles. The K-ion diffusion coefficient of **j** Se/N-3DMpC and **k** Se-3DMpC calculated by the GITT during potassiation and depotassiation processes

Figure 4a–c displays the ex situ Raman spectra during the second discharging/charging cycle and the corresponding I_D_/I_G_ ratio. The intensity of D and G bands gradually weakens during the discharging process owing to the formation of KC_x_ which could narrow the depth of optical skin [43]. In the depotassiation process, the peaks intensity could return to the pristine state, suggesting the excellent reversibility of the architecture. At the same time, compared with the pristine one, the *I*_D_/*I*_G_ value of Se/N-3DMpC increased after potassiation (Fig. 4c), which derives from the augment of disordered state with the insertion of K^+^ [49]. With the extraction of K^+^, the value decreases again, indicating that the structure has good reversibility. Additionally, ex situ XRD showed that the (002) peak slightly shifted negatively during the discharging process, and can be returned to the original position during the charging process, revealing that the insertion of K^+^ would only cause the mild expansion of interlayer spacing owing to the enlarged carbon interlayer distance (Fig. 4d) [50]. This is consistent with the phenomenon observed in ex situ (HR)TEM and SEM measurements (Figs. 4e–h and S27). In addition, the morphology of the Se/N-3DMpC electrode did not change significantly (Fig. S27), and the interlayer spacing was slightly enlarged to 0.42 nm (Fig. 4e). The EDS mappings manifest that K, Se, N, and C distribute uniformly over the electrode materials (Figs. 4f–g and S27), which further confirms potassium ions are uniformly embedded in Se/N-3DMpC electrode. The interlamellar distance of Se/N-3DMpC returned to 0.41 nm upon depotassiation (Fig. 4h), implying the expansion of interlayer spacing caused by chemical bonds in Se/N-3DMpC could withstand repeated insertion and extraction of potassium ions without drastic changes. The *ex*-*situ* XPS spectra were analyzed to study the related properties of the Se/N-3DMpC electrode during the electrochemical process. In the first fully discharged state, the peaks of Se 3d_5/2_ and Se3d_3/2_ decrease and shift negatively, which indicates the strong interaction between doped Se and K (Fig. S28a). In the first fully charged state, these peaks show positive displacement, and the peak positions almost remain unchanged after 200 cycles, which verifies the stable structure of Se/N-3DMpC (Fig. S28a). Moreover, the high-resolution spectrum of N 1 s of Se/N-3DMpC electrode shifted negatively at discharged state and can be returned to the original position at fully charged state, further proving the excellent reversibility of the Se/N-3DMpC electrode (Fig. S28b).

The galvanostatic intermittent titration technique (GITT) was employed to further investigate the potassium-ion diffusion coefficient (D_k_) in Se/N-3DMpC and Se-3DMpC during charging/discharging process (Figs. 4i and S29) [51]. The *D*_k_ values in Se/N-3DMpC electrode are higher than those of Se-3DMpC electrode at all potentials during cycling (Fig. 4j, k). The enhanced diffusion rate of potassium ions can be rationally attributed to the expanded ionic diffusion channels and porous network. To further get deeper atomic insight into the fast kinetics for the Se/N-3DMpC, the diffusion barrier energies (*E*_b_) of K^+^ in the synthesized samples have been investigated by DFT method (Fig. S30). The diffusion path of potassium ion between the two representative neighboring hollow sites in the pure-C, N-pC, Se-3DMpC, and Se/N-3DMpC is shown in Figs. S31–S34, respectively. It should be noted that the Se/N-3DMpC delivers the lowest diffusion energy barrier (0.27 eV) compared with that of Se-3DMpC (0.32 eV), N-pC (0.51 eV), and pure-C (0.54 eV), confirming the introduction of Se and N dopants conduces to decrease the diffusion resistance of K^+^.

### Structure and Electrochemical Performance of A-Se/N-3DMpC Cathode

The Se/N-3DMpC was then activated by KOH in order to increase the SSA so that it can adsorb more anions. The as-obtained activated Se/N-3DMpC (A-Se/N-3DMpC) was employed as the capacitor-type cathode of the PIHCs. The 3D hierarchical porous morphology of A-Se/N-3DMpC is well maintained, and a plenty of pores are evenly dispersed in the hierarchical porous architecture (Fig. S35a–e). The EDS mappings and XPS analysis confirm that the heteroatoms of Se and N still remain in the A-Se/N-3DMpC (Figs. S35f–i and S36a–c), which could furnish abundant active sites for adsorption/desorption of anions and afford additional pseudocapacitive energy storage. Furthermore, the DFT calculation suggests that adsorption energy of anions on the A-Se/N-3DMpC is stronger than that on the pure carbon, indicating that Se/N co-doping conduces to the adsorption of anions on the carbon (Fig. S37a, b). Also, charge density difference was conducted to analyze the transferred charges between adsorbed PF_6_^−^ and the different carbon materials. More transferred charges can be observed between adsorbed PF_6_^−^ and Se/N co-doped carbon, indicating that Se/N co-doping is conducive to improving the adsorption ability of anions. The N_2_ adsorption–desorption report demonstrates that the SSA of A-Se/N-3DMpC is as high as 1453.75 m^2^ g^−1^ (Fig. S36d), much higher than that of the Se/N-3DMpC, implying that the A-Se/N-3DMpC can provide a large number of active sites for anion adsorption. The CV profiles of the A-Se/N-3DMpC present a quasi-rectangular shape at diversity sweep rates (Fig. S38a), well reflecting a capacitive behavior [52]. Additionally, the rate performance of the A-Se/N-3DMpC cathode exceeds that of the AC previously reported, suggesting an excellent electrochemical reversibility (Fig. S38b, c) [37]. Moreover, 82.6% of the capacity retention with nearly 100% Coulombic efficiency of A-Se/N-3DMpC is achieved after 4000 cycles at 1.0 A g^−1^ (Fig. S38d), which makes it be an ideal cathode material for high-performance PIHCs.

### Electrochemical Performance Tests in PIHCs

**Fig. 5:**
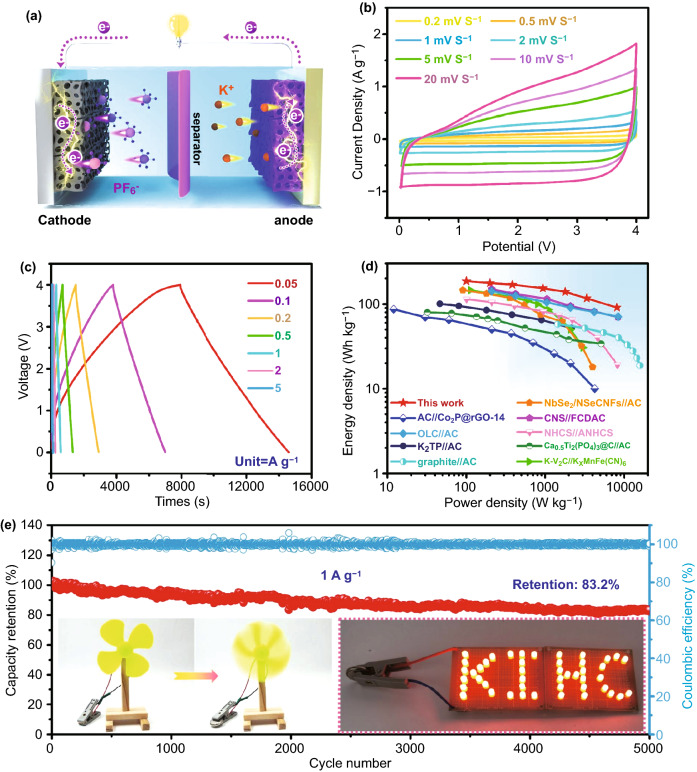
**a** Schematic illustration of the A-Se/N-3DMpC//Se/N-3DMpC PIHCs device. **b** CV curves at various sweep rates and **c** charge/discharge curves at diversity current density of the A-Se/N-3DMpC//Se/N-3DMpC PIHCs. **d** Comparison of the energy/power density of the A-Se/N-3DMpC//Se/N-3DMpC PIHCs with the reported works. **e** Long-term cycle performance of the PIHCs at 1 A g^−1^. Inset: Photograph of mini-windmill and LED powered by the A-Se/N-3DMpC//Se/N-3DMpC PIHCs

A full-cell PIHC was assembled with A-Se/N-3DMpC as the capacitive-type cathode and Se/N-3DMpC as the battery-type anode (Figs. 5a and S39). Figure 5a shows the schematic diagram of the A-Se/N-3DMpC//Se/N-3DMpC PIHC, where K^+^ is inserted/extracted into/from the Se/N-3DMpC anode, while PF_6_^−^ is adsorbed/desorbed on the surface of A-Se/N-3DMpC cathode during charging/discharging. In order to obtain the stable electrochemical performance for PIHCs, the operating voltage was optimized to be in the window range of 0.01–4.0 V (Fig. S40). The PIHCs exhibited a near-rectangular shape CV curves (Fig. 5b) and quasi-triangular shape GCD originates from the coupling effect of Faradaic and non-Faradaic processes (Fig. 5c) [53, 54]. Moreover, the A-Se/N-3DMpC//Se/N-3DMpC PIHCs displayed the highest energy density of 186 Wh kg^−1^ (at power density of 100 W kg^−1^) and can maintain an energy density of 91 Wh kg^−1^ even at a high power density of 8100 W kg^−1^, which surpass most of the previously reported PIHCs (Fig. 5d) [17, 20, 24, 34, 53, 55–58]. The excellent energy and power densities output of the PIHCs can bridge the gap between high-energy rechargeable batteries and high-power supercapacitors (Fig. S41). Moreover, the optimized A-Se/N-3DMpC//Se/N-3DMpC PIHCs could retain 83.2% of capacity after 5000 cycles at a current density of 1 A g^−1^ (Fig. 5e). Meanwhile, the cycling stability of the A-Se/N-3DMpC//Se/N-3DMpC PIHCs exceeds most of the previously reported PIHCs (Table S3). Notably, this A-Se/N-3DMpC//Se/N-3DMpC PIHCs device can easily actuate a mini-windmill and light up a “KIHC” logo including 46 light-emitting diodes (LEDs) in parallel (in inset of Fig. 5e), further demonstrating the potential practical application of the PIHCs.

According to the above analysis, the outstanding electrochemical properties of PIHCs using Se/N-3DMpC and A-Se/N-3DMpC electrodes can be legitimately ascribed to their distinctive structure and component advantages: (1) 3D hierarchical porous architecture can afford adequate contact area between the electrolyte and the active sites to facilitate the transport of electrolyte and ions; (2) 3D framework carbon potentially provides more capability by an electrical double-layer capacitance, which could reinforce the K^+^ storage capacity for PIHCs; (3) the enlarged interlayer space is conducive to the intercalation/extraction of large-size K ions; and (4) the numerous defects and additional active sites caused by the co-doping of Se and N can be beneficial to improve the adsorption capability of cations/anions, resulting in the rapidly electrochemical reaction kinetics for anode and improved capacity for cathode.

## Conclusions

In summary, a reliable and versatile self-sacrificial template method was developed to fabricate Se and N co-doped 3D macroporous carbon (Se/N-3DMpC). Benefiting from the advantages of 3D hierarchical porous architecture, enlarged interlayer space and abundant heteroatoms doping, Se/N-3DMpC exhibited a high reversible capacity and attractive kinetic property for potassium-ion storage. Meanwhile, the profusion active sites and hierarchical porous structure as well as the ultrahigh surface area of A-Se/N-3DMpC endow it with high specific capacitance as cathode of PIHCs. Taking advantages of the Se/N-3DMpC anode and the A-Se/N-3DMpC cathode, we demonstrate a high-performance PIHC that was capable of delivering high energy density of 91 Wh kg^−1^ at a high power density of 8100 W kg^−1^ and exhibit outstanding cycling stability. This work may open a new avenue for fabricate advanced materials available for a variety of applications.

## Supplementary Information

Below is the link to the electronic supplementary material.

Supplementary file1 (PDF 4130 kb)
