# Supplementary material for: A General Self-Sacrifice Template Strategy to 3D Heteroatom-Doped Macroporous Carbon for High-Performance Potassium-Ion Hybrid Capacitors
Source: Nanomicro Lett. 2021 May 29;13:131. doi: 10.1007/s40820-021-00659-7 (PMC8163926; doi:10.1007/s40820-021-00659-7)
Supplement: Supplementary file 1 — Supplementary file1 (PDF 4130 kb) [file 40820_2021_659_MOESM1_ESM.pdf]

Supporting Information for

A General Self-Sacrifice Template Strategy to 3D Heteroatom-Doped Macroporous Carbon for High-Performance Potassium-Ion Hybrid Capacitors

Junwei Li[1], Xiang Hu[1, *], Guobao Zhong[1], Yangjie Liu[1], Yaxin ji[1], Junxiang Chen[1], Zhenhai Wen[1, *]

[1]CAS Key Laboratory of Design and Assembly of Functional Nanostructures, and Fujian Provincial Key Laboratory of Nanomaterials, Fujian Institute of Research on the Structure of Matter, Chinese Academy of Sciences, Fuzhou, Fujian 350002, P. R. China

*Corresponding authors. E-mail: huxiang@fjirsm.ac.cn (Xiang Hu),

wen@fjirsm.ac.cn (Zhenhai Wen)

Supplementary Figures and Tables

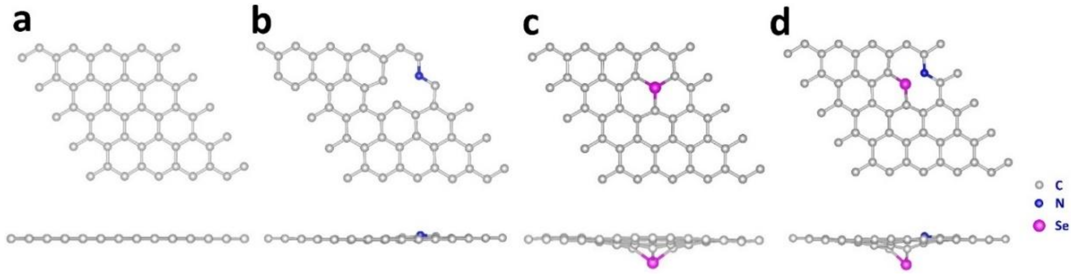

Fig. S1 Top and side view of the optimized **(a)** pure carbon, **(b)** N-doped carbon, **(c)** Se-doped carbon and **(d)** Se/N co-doped carbon

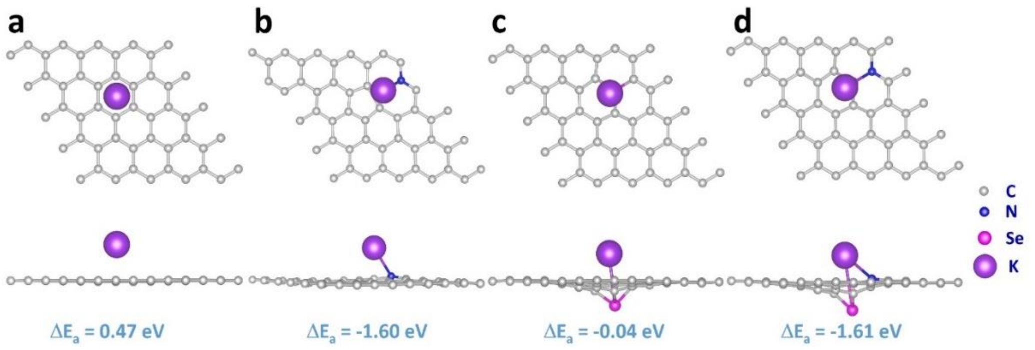

Fig. S2 Top and side view of the simulations for one K ion adsorbed on the **(a)** pure carbon, **(b)** N-doped carbon, **(c)** Se-doped carbon and **(d)** Se/N co-doped carbon as well as corresponding ΔE_a

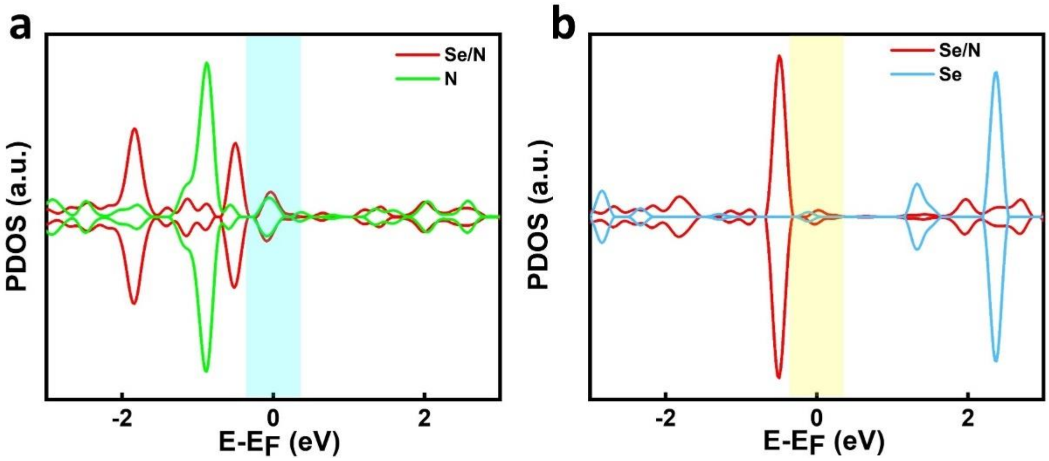

Fig. S3 (a) the p orbital partial density of states (PDOS) of the nitrogen atom in the Se/N co-doped carbon and N doped carbon. **(b)** the p orbital PDOS of the selenium atom in the Se/N co-doped carbon and Se doped carbon

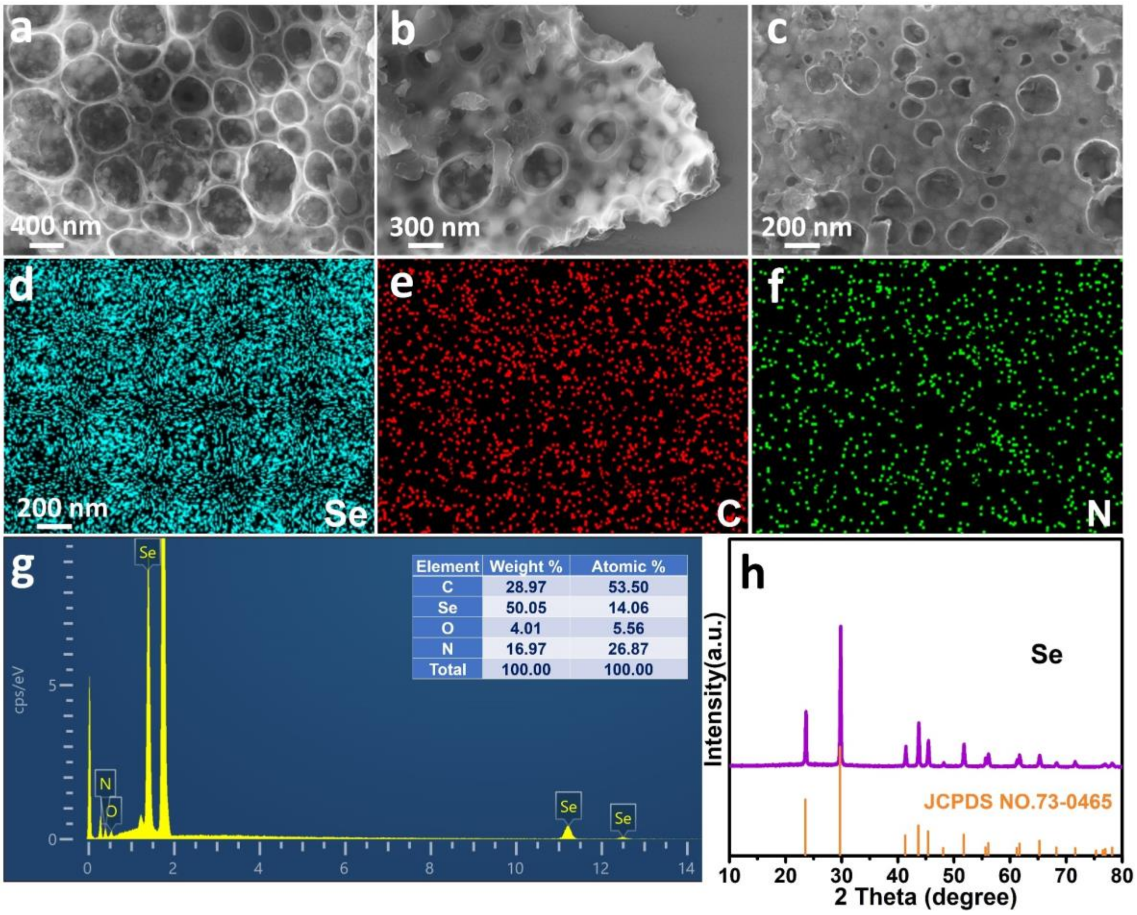

Element	Weight %	Atomic %
C	28.97	53.50
Se	50.05	14.06
O	4.01	5.56
N	16.97	26.87
Total	100.00	100.00

Fig. S4 (a-c) FESEM images and **(d-f)** corresponding elemental mapping images, **(g)** EDS spectra and **(h)** XRD pattern of the Se@C-precursor at 200 ℃

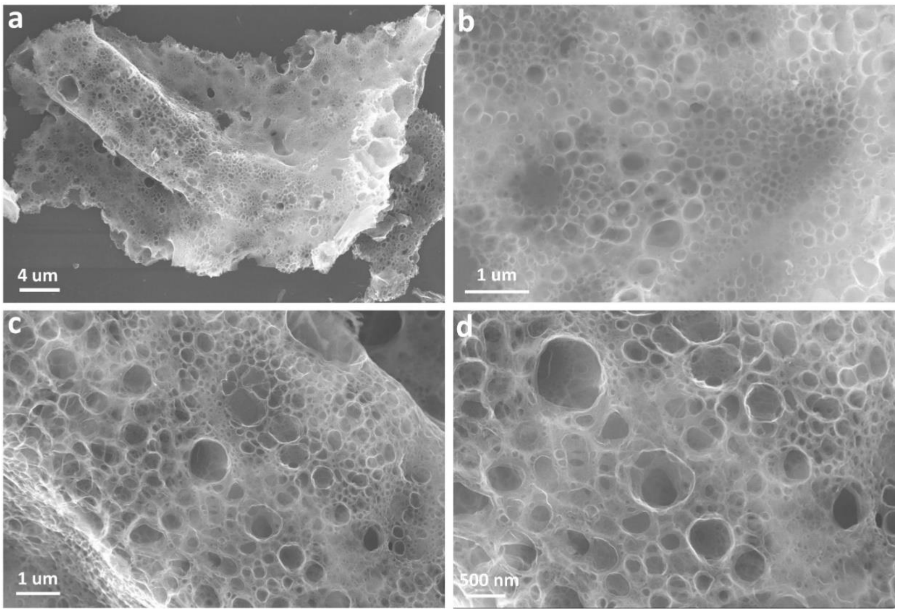

Fig. S5 (a-d) FESEM images of the Se/N-3DMpC at different magnifications

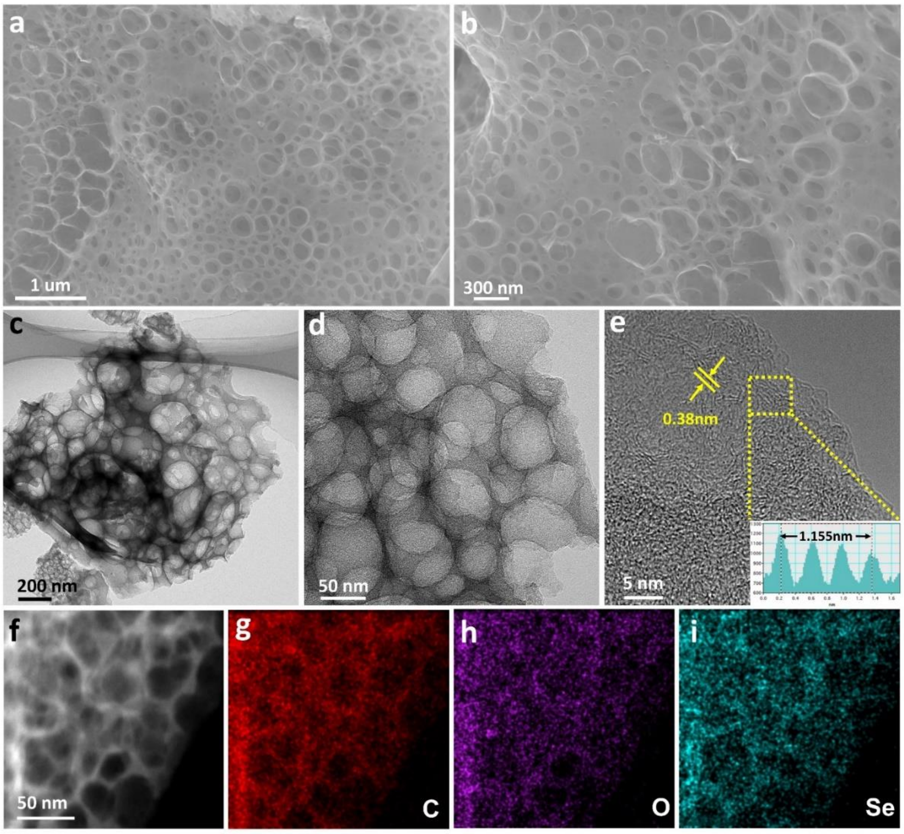

Fig. S6 (a, b) FESEM images, **(c, d)** TEM images, **(e)** HRTEM image and **(f-i)** HAADF-STEM image and elemental mappings of the Se-3DMpC

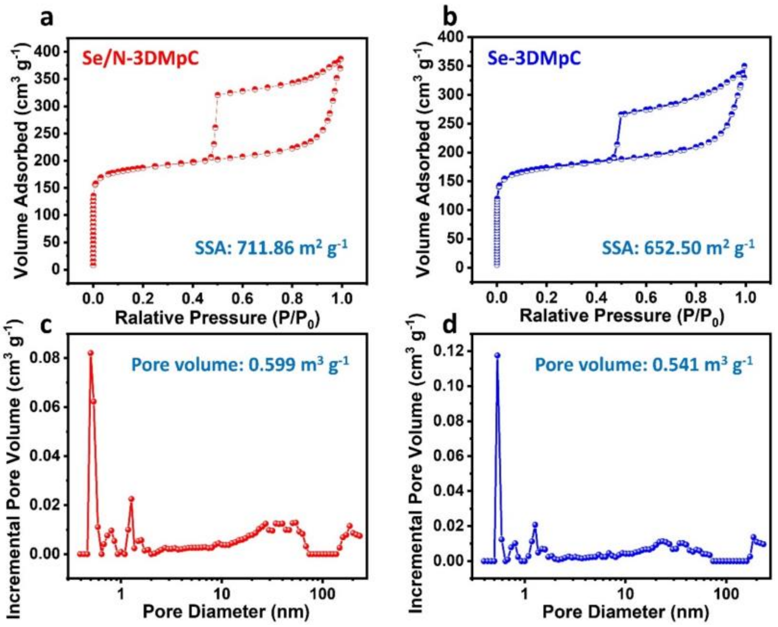

Fig. S7 Nitrogen adsorption-desorption isothermal curves of **(a)** Se/N-3DMpC and **(b)** Se-3DMpC. Pore size distribution of **(c)** Se/N-3DMpC and **(d)** Se-3DMpC

Figure S7 shows the nitrogen absorption-desorption isothermal curve for the Se/N-3DMpC and Se-3DMpC, which combined type-I and type-IV isotherms with an H4 hysteresis loop desorption. The Se/N-3DMpC displays a high SSA of 711.86 m^2 g^{-1}, which is higher than that of Se-3DMpC (652.50 m^2 g^{-1}). The corresponding porosity distribution curve is shown in Figure S7c-d, indicating the Se/N-3DMpC and Se-3DMpC have hierarchical micro-meso-macro porous structure, which are well in accordance with the TEM and SEM results.

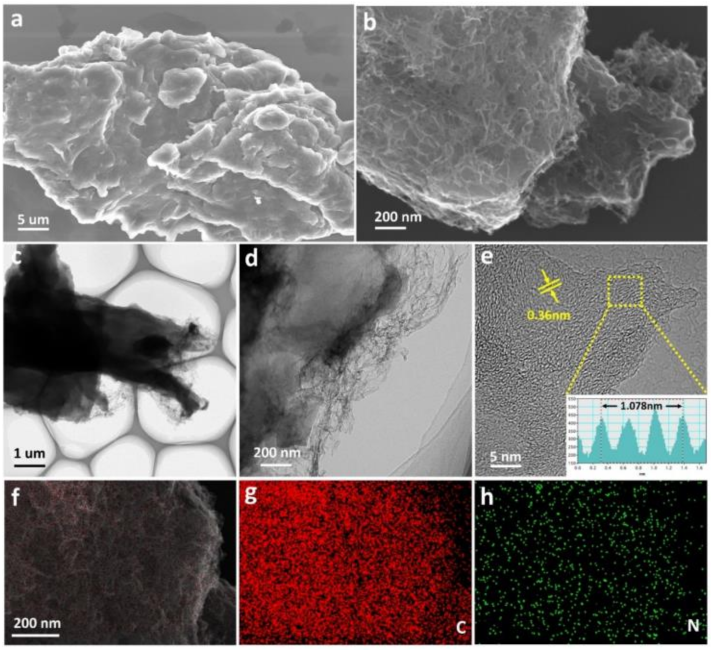

Fig. S8 (a, b) FESEM images, **(c, d)** TEM images, **(e)** HRTEM image and **(f-h)** FESEM image and corresponding elemental mappings of the N-pC

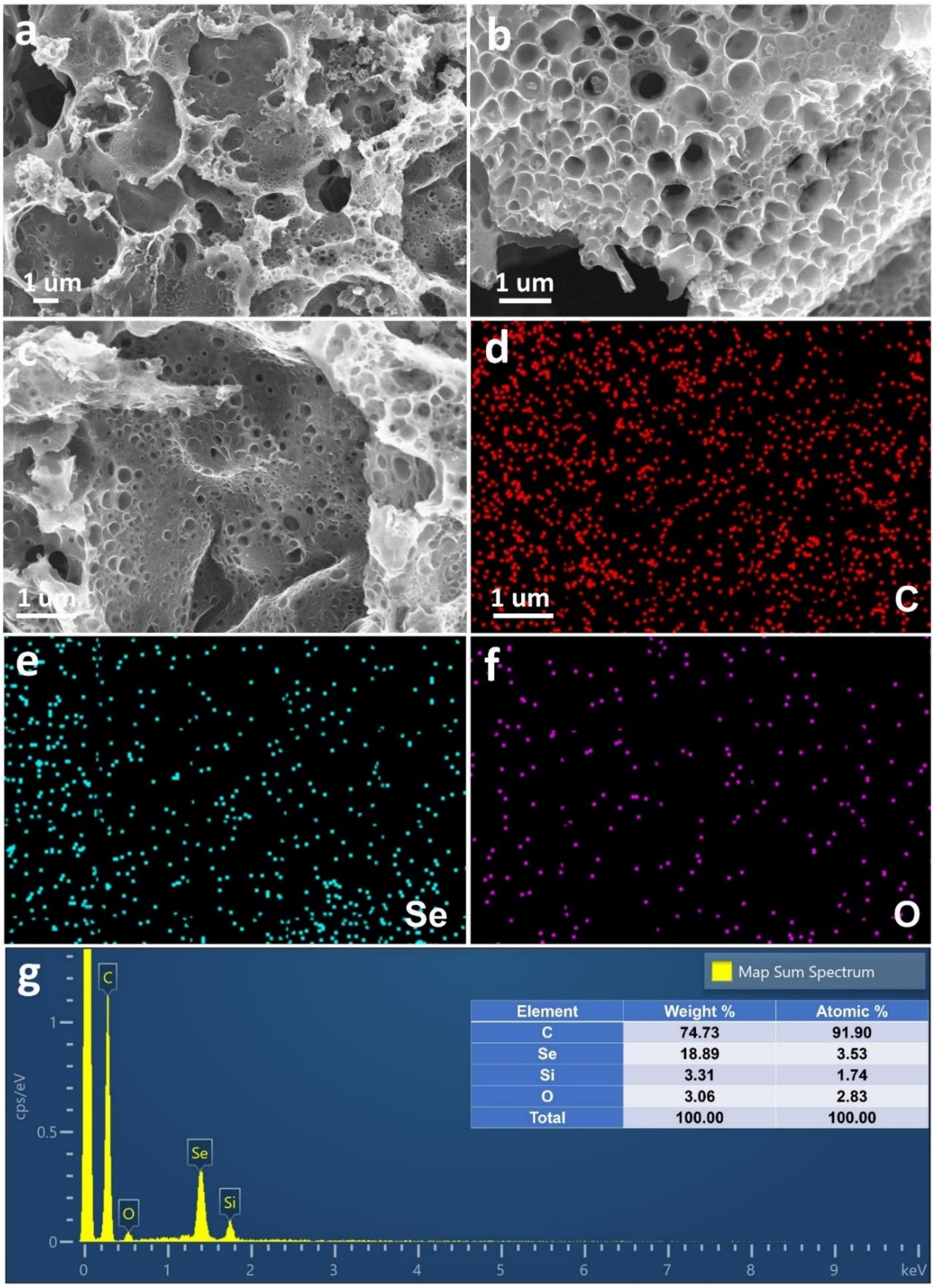

Element	Weight %	Atomic %
C	74.73	91.90
Se	18.89	3.53
Si	3.31	1.74
O	3.06	2.83
Total	100.00	100.00

Fig. S9 (a-c) FESEM images, **(d-f)** corresponding elemental mappings and **(g)** EDS spectra of the starch-based Se-doped 3DMpC

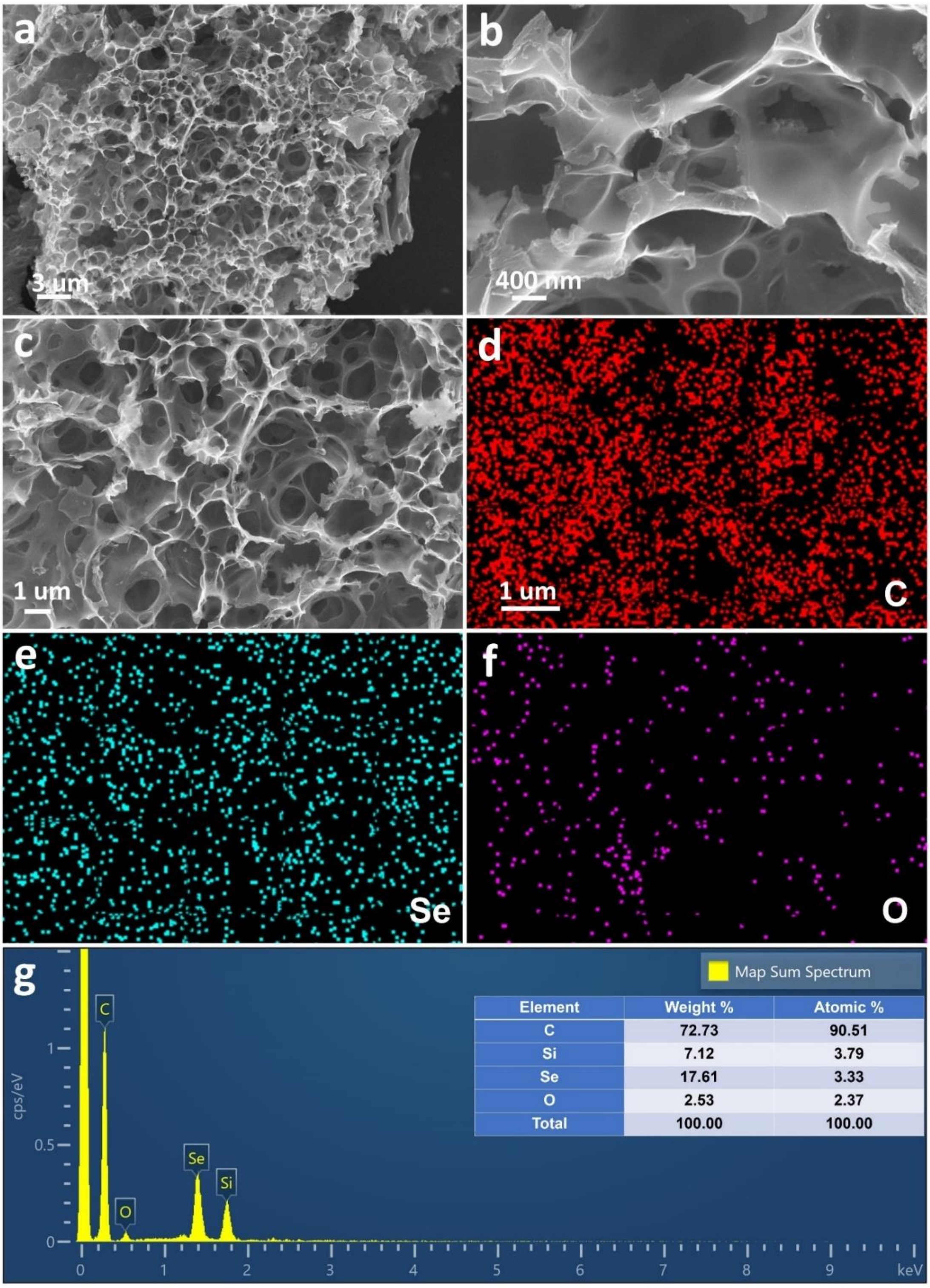

Fig. S10 (a-c) FESEM images, **(d-f)** corresponding elemental mappings and **(g)** EDS spectra of the glucose-based Se-doped 3DMpC.

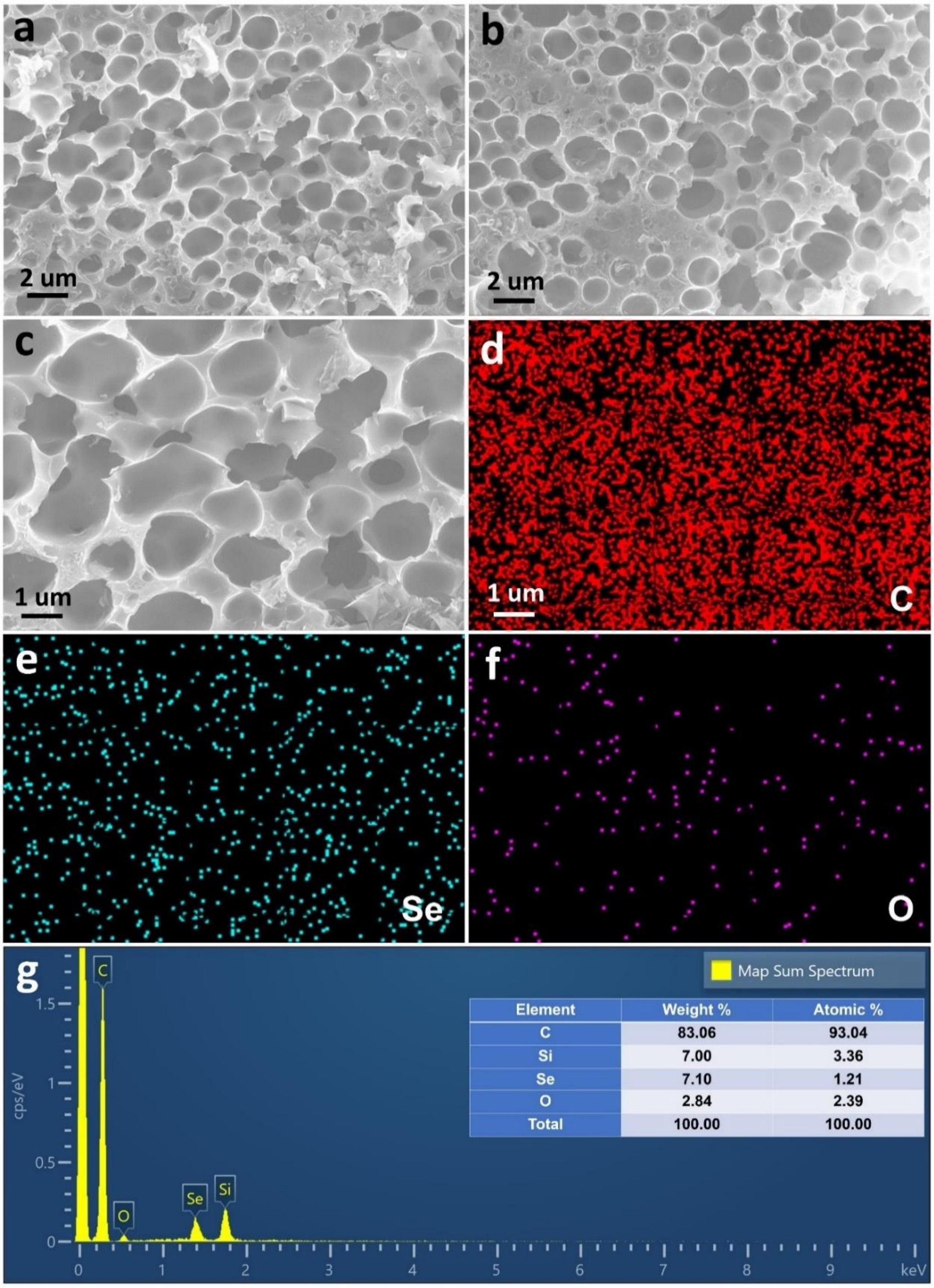

Fig. S11 (a-c) FESEM images, **(d-f)** corresponding elemental mappings and **(g)** EDS spectra of the PVP-based Se-doped 3DMpC

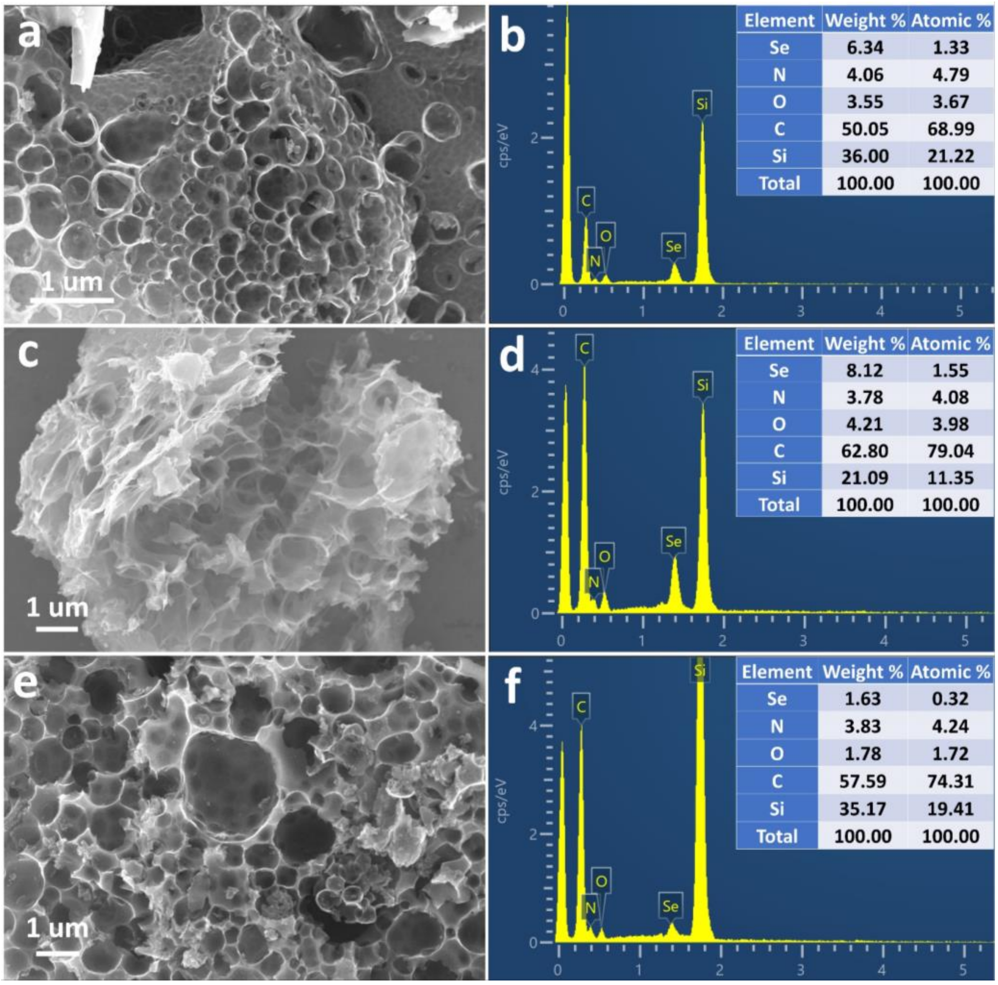

Fig. S12 A series of Se/N co-doped 3DMpC can be obtained from urea and different carbon sources. **(a)** FESEM image and **(b)** EDS spectra of the starch-based Se/N co-doped 3DMpC. **(c)** FESEM image and **(d)** EDS spectra of the glucose-based Se/N co-doped 3DMpC. **(e)** FESEM image and **(f)** EDS spectra of the PVP-based Se/N co-doped 3DMpC

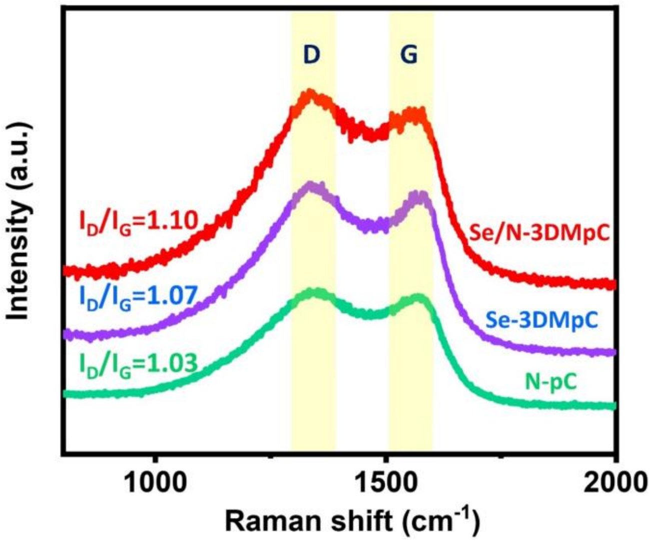

Fig. S13 Raman spectra of the Se/N-3DMpC, Se-3DMpC and N-pC

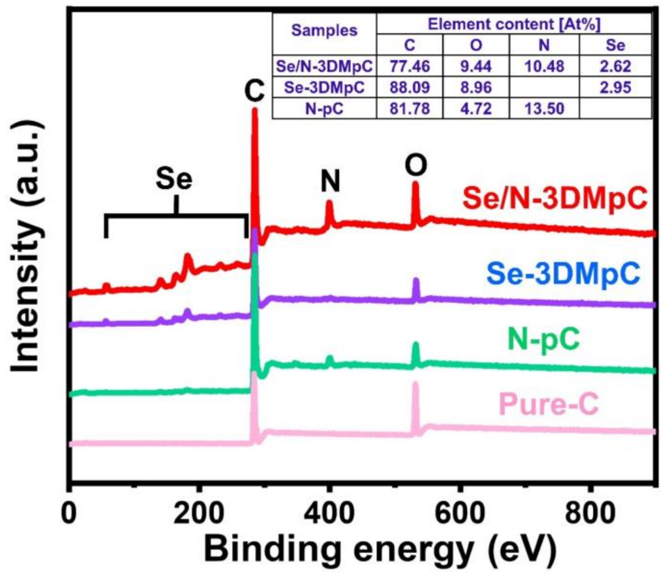

Fig. S14 XPS spectra of Se/N-3DMpC, Se-3DMpC, N-pC and pure-C

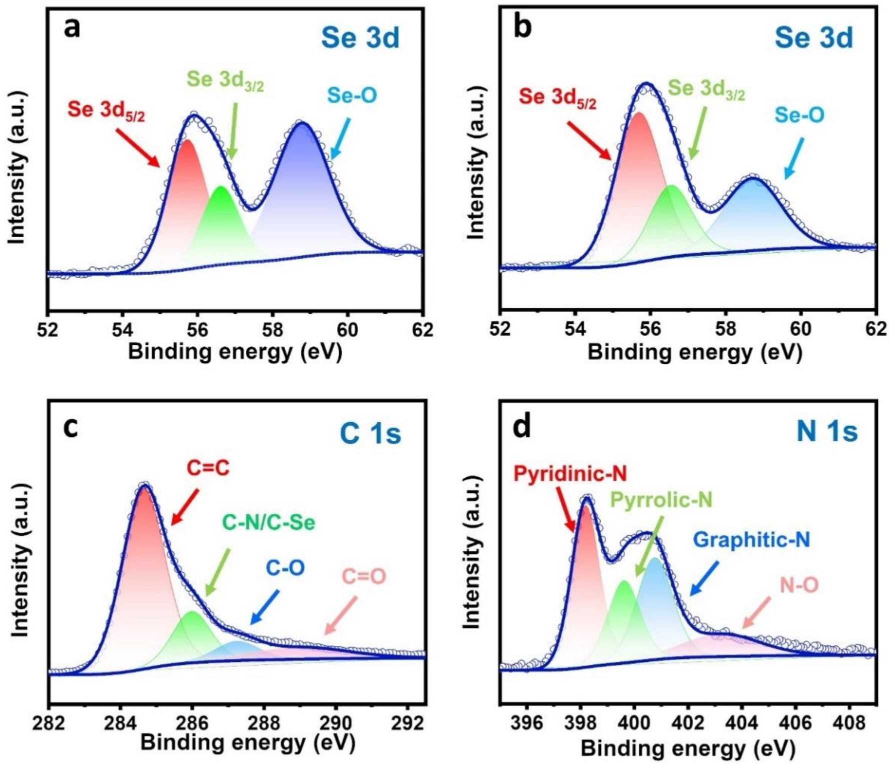

Fig. S15 High-resolution Se 3d XPS spectra of **(a)** Se/N-3DMpC and **(b)** Se-3DMpC. **(c)** High-resolution C 1s and **(d)** N 1s XPS spectra of the Se/N-3DMpC

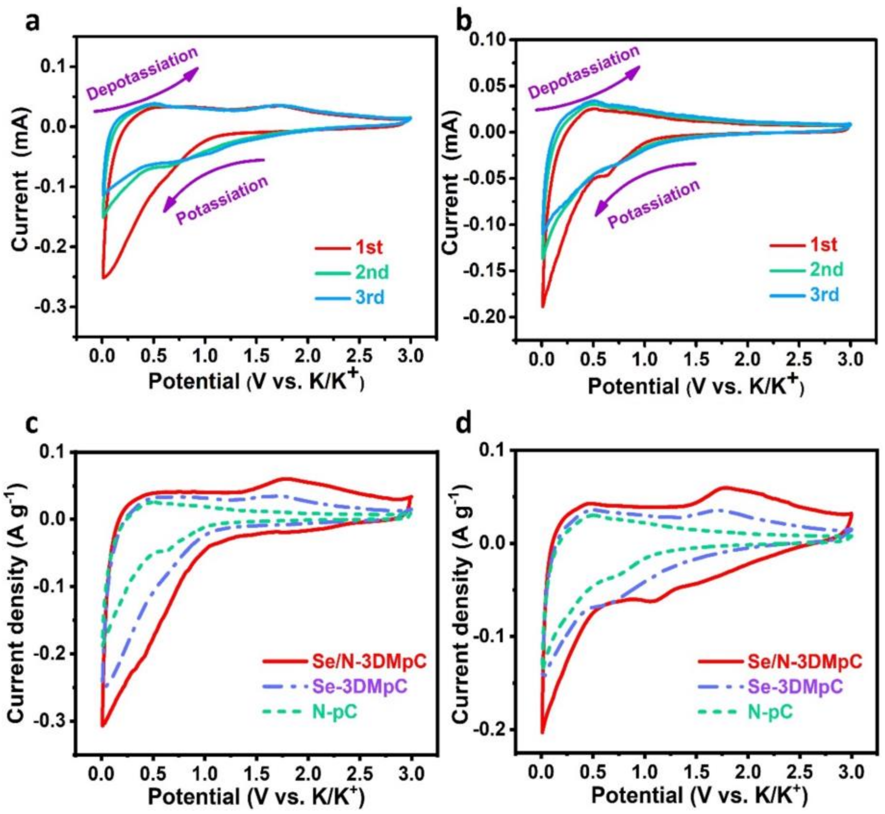

Fig. S16 The CV curves at a sweep rate of 0.1 mV s^{-1} of **(a)** Se-3DMpC and **(b)** N-pC. CV curves of the Se/N-3DMpC, Se-3DMpC and N-pC electrodes at a scan rate 0.1 mV s^{-1} for the **(c)** 1st cycle and **(d)** 2nd cycle

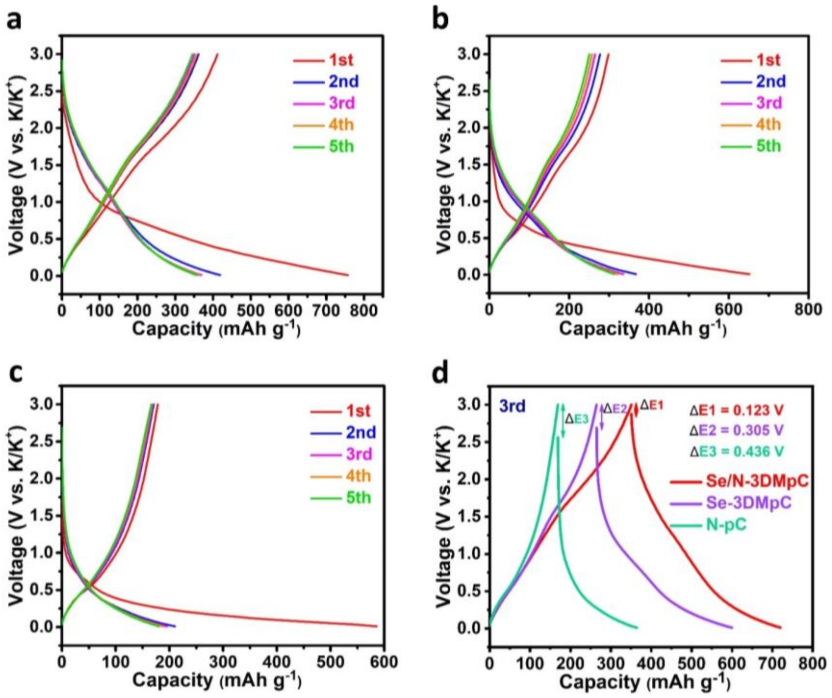

Fig. S17 The galvanostatic charge-discharge curves of **(a)** Se/N-3DMpC, **(b)** Se-3DMpC and **(c)** N-pC electrodes at a current density of 0.2 A g^{-1}. **(d)** The third charge-discharge curves of Se/N-3DMpC, Se-3DMpC and N-pC

The low ICE of 54.3% for the Se/N-3DMpC electrode might be due to the electrolyte decomposition and subsequent formation of SEI. Choosing a suitable electrolyte and binder (such as ether-based electrolyte and carboxymethyl cellulose) are good ways to improve the ICE value in carbon-based material in our future research.

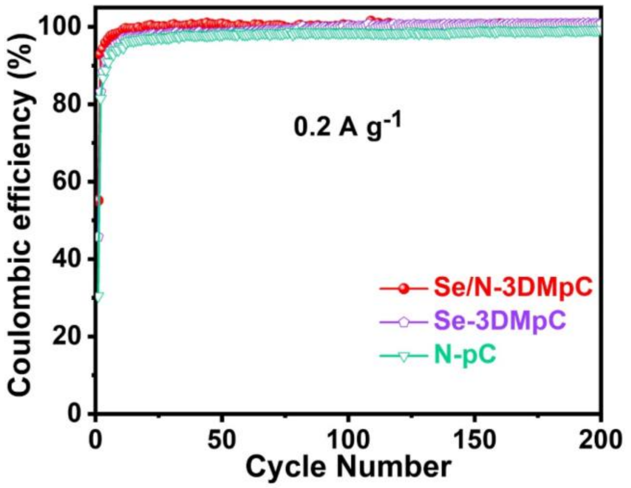

Fig. S18 Coulombic efficiency of the Se/N-3DMpC, Se-3DMpC and N-pC electrodes for PIBs at current density of 0.2 A g^{-1} for 200 cycles

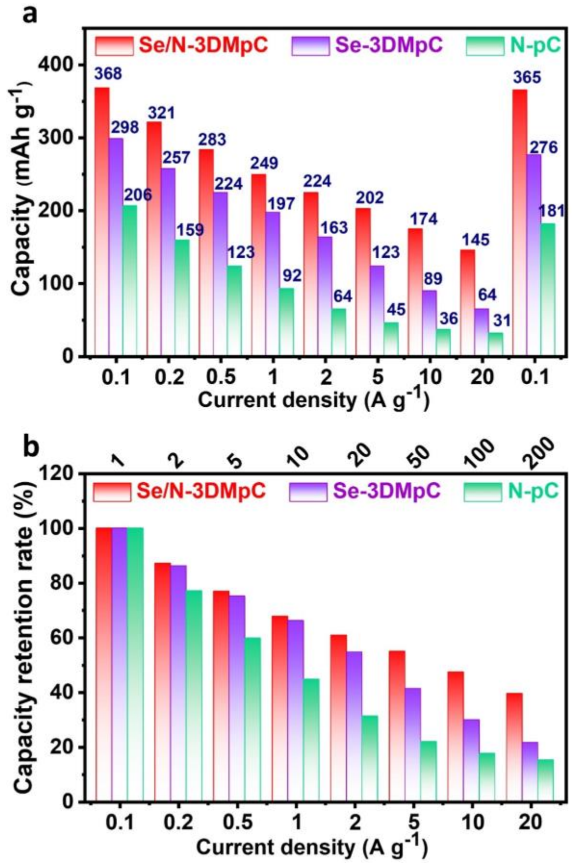

Fig. S19 (a) The average capacities and **(b)** capacity retention rate of the Se/N-3DMpC, Se-3DMpC and N-pC at different current densities for PIBs corresponding to Fig. 3c

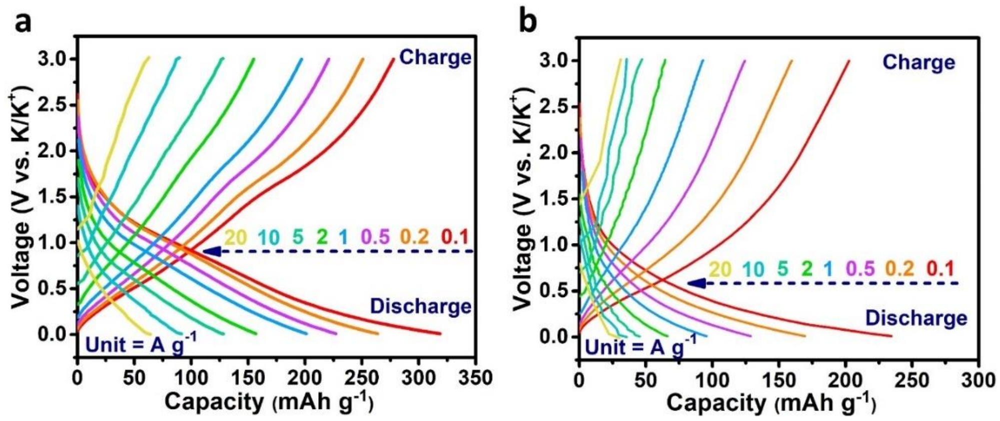

Fig. S20 Charge-discharge curves of **(a)** Se-3DMpC and **(b)** N-pC electrodes at various current densities

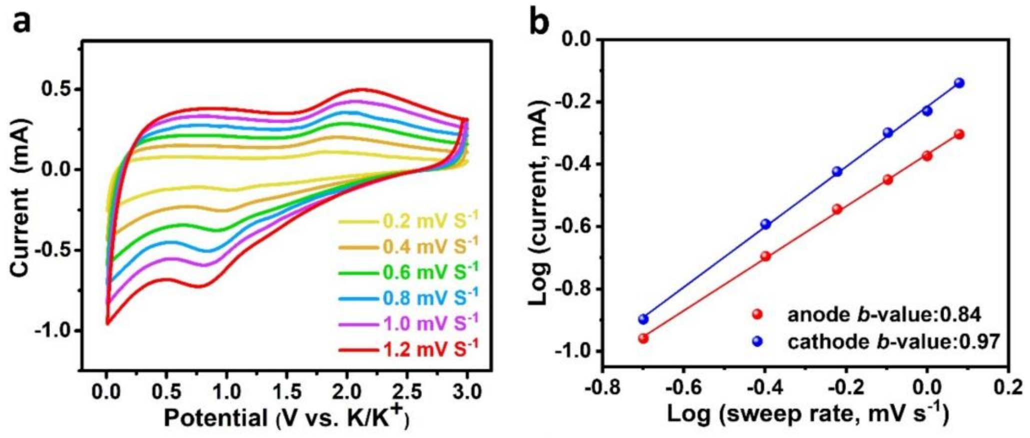

Fig. S21 (a) CV profiles at various sweep rates from 0.2 to 1.2 mV s^{-1}, **(b)** b value determination of the Se/N-3DMpC electrode

As shown in Fig. S21a, the shape of the CV profiles is commendably retention with increasing the sweep rate. The potassium storage kinetics of all electrodes can be evaluated according to equation : $i = a\, v^{b}$, where the measured current (i) obeys a power-law relationship with the sweep rate (v), a and b are adjustable parameters, and the value of b can be calculated from the slope by plotting log (i) against log (v). Generally, when the value of b is 0.5 or 1, the charge storage process is dominated by the diffusion-controlled or surface-controlled, respectively. As displayed in Fig. S21b, the calculated b values are above 0.84 and 0.97 for anodic and cathodic peaks, suggesting a prompt kinetics dominated by the capacitive behavior of surface-controlled on account of the abundant Se and N dopants. On the contrary, because there are fewer functional groups that can conducive to increase the pseudocapacitance of the materials, all the b values of N-pC and Se-3DMpC are inferior than those of Se/N-3DMpC (Figs. S21a-b, S22a-b and S23a-b), suggesting that Se/N-3DMpC electrode has a faster reaction kinetics. According to the equation $i(V)=k_1v+k_2v^{1/2}$, the contribution of surface-controlled (k_1v) and diffusion-controlled ($k_2v^{1/2}$) at various sweep rates can be further quantitatively separated.

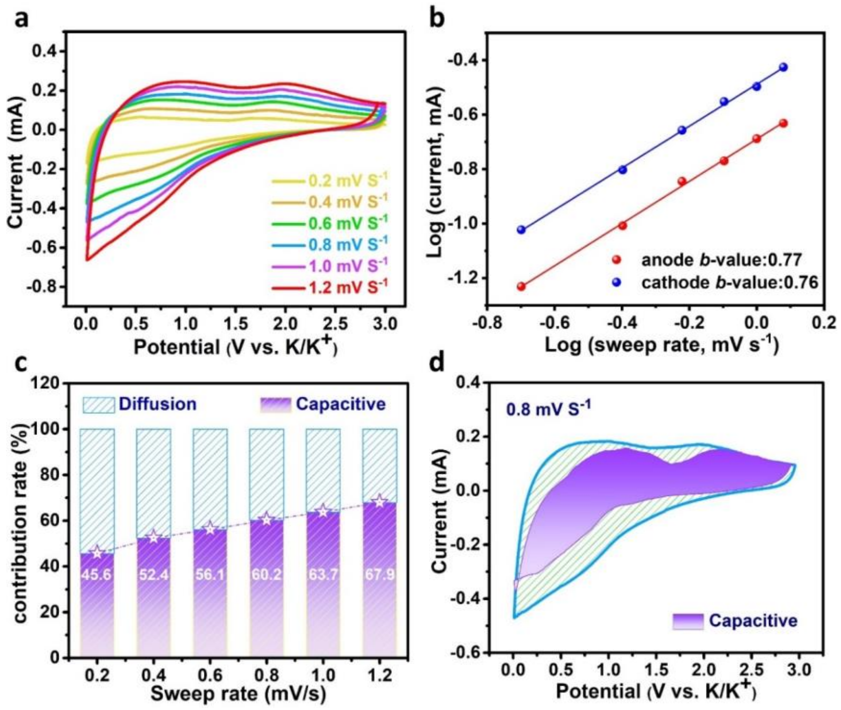

Fig. S22 (a) CV profiles at various sweep rates from 0.2 to 1.2 mV s^{-1}, **(b)** b value determination, **(c)** contribution of the surface process and **(d)** capacitive contribution at 0.8 mV s^{-1} of the Se-3DMpC

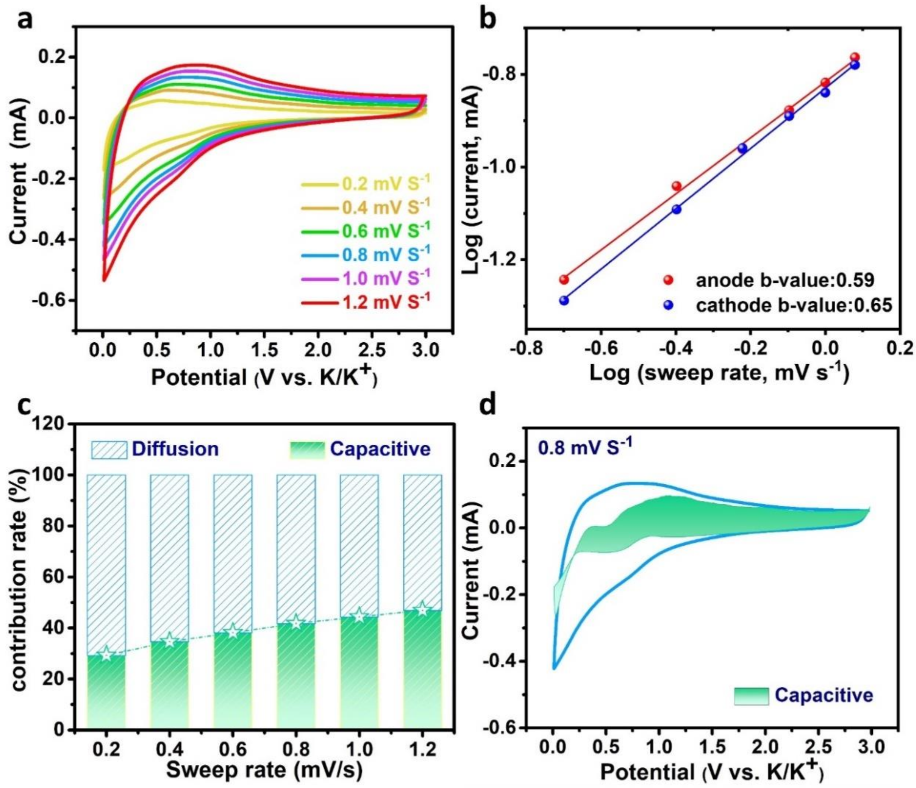

Fig. S23 (a) CV profiles at various sweep rates from 0.2 to 1.2 mV s^{-1}, **(b)** b value determination, **(c)** contribution of the surface process and **(d)** capacitive contribution at 0.8 mV s^{-1} of the N-pC

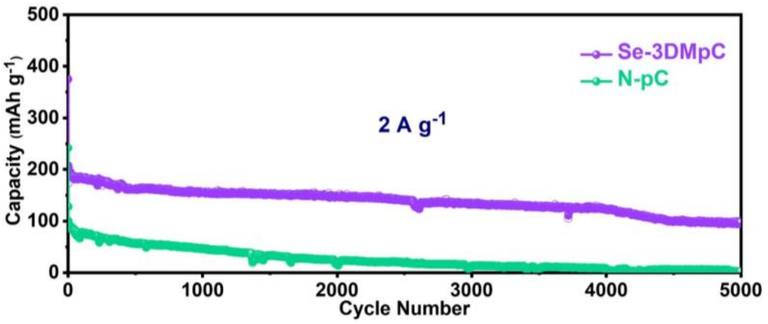

Fig. S24 Long-term cycling performance of the Se-3DMpC and N-pC at a current density of 2.0 A g^{-1}

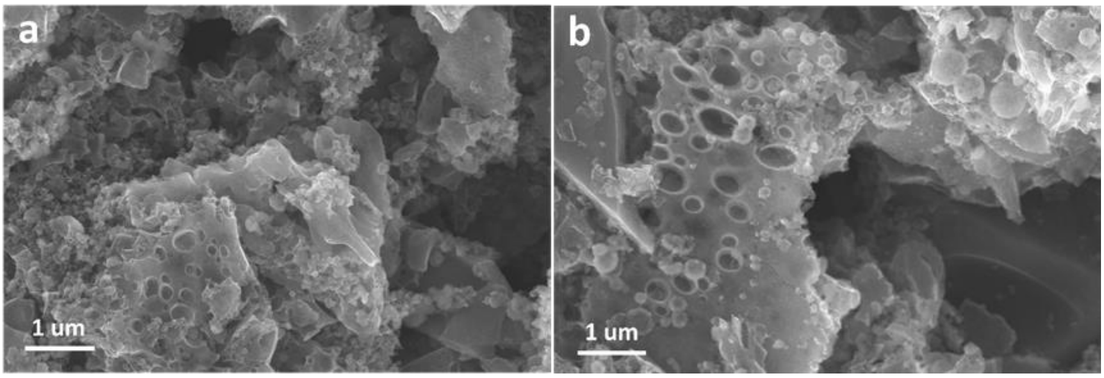

Fig. S25 (a, b) FESEM images of Se/N-3DMpC electrode after 5000 cycles for PIBs

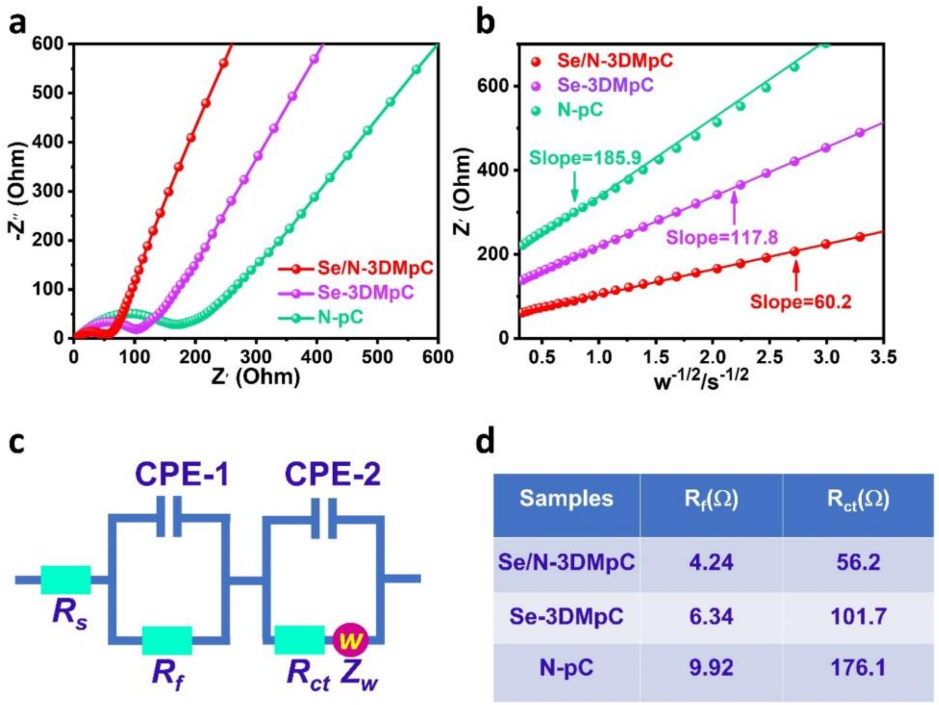

Samples	$R_f(\Omega)$	$R_{ct}(\Omega)$
Se/N-3DMpC	4.24	56.2
Se-3DMpC	6.34	101.7
N-pC	9.92	176.1

Fig. S26 (a) Electrochemical impedance spectra of the Se/N-3DMpC, Se-3DMpC and N-pC electrodes and **(b)** linear fits of the Z` versus $\omega^{-1/2}$ (ω =2πf) in the low-frequency region. **(c)** the equivalent circuit simulation. **(d)** The EIS fitting data of Se/N-3DMpC, Se-3DMpC and N-pC electrodes

Fitting the corresponding equivalent circuit shows an ohmic contact resistance (R_s) from the electrolyte and electrode, the resistance (R_f) of the potassium ions through SEI film and charge-transfer resistance (R_{ct}). Zw represents the Warburg impedance of K^+ diffusion in the electrode material related to the slope of the straight line in the low-frequency region. CPE-1 and CPE-2 correspond to surface capacitance and double layer capacitance respectively.

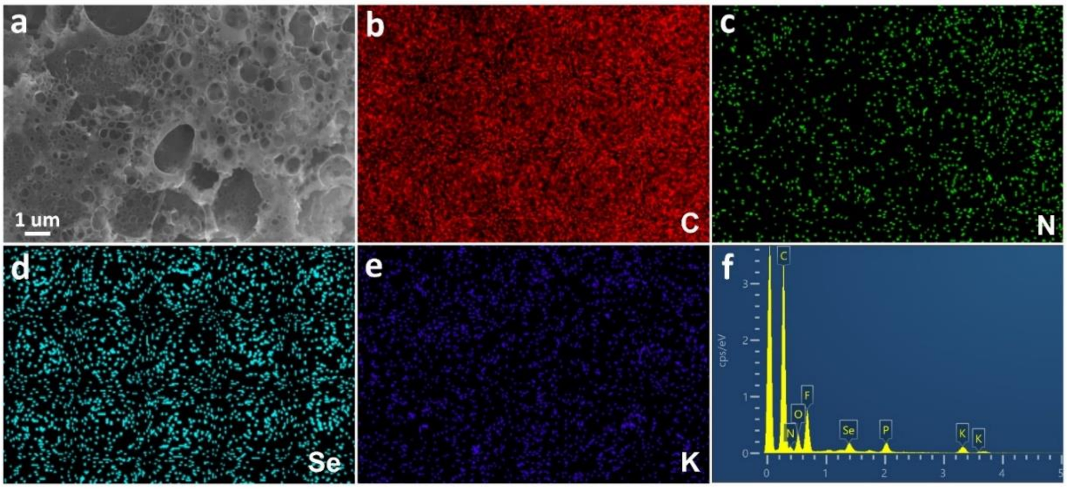

Fig. S27 (a) FESEM images, **(b-e)** corresponding elemental mappings and **(f)** EDS spectra of the Se/N-3DMpC at the first potassiation state

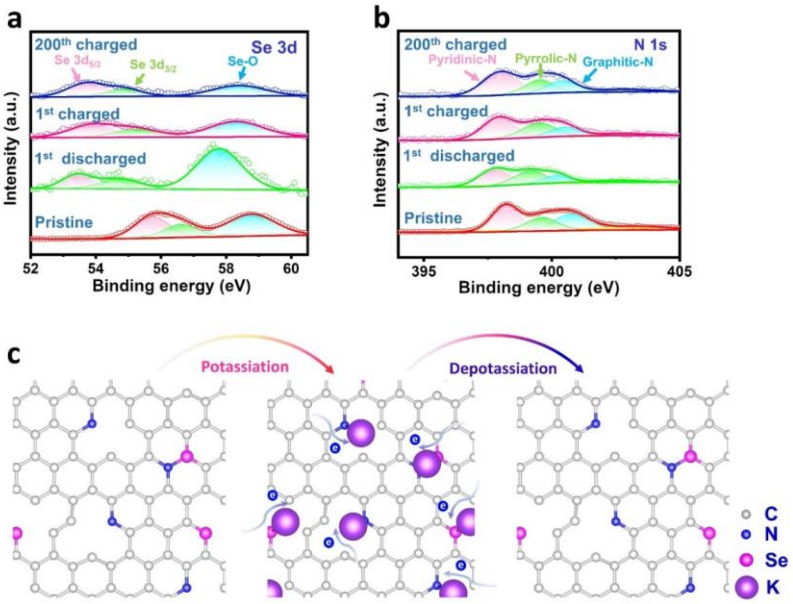

Fig. S28 The ex-situ high-resolution XPS spectra of **(a)** Se 3d and **(b)** N 1s for the Se/N-3DMpC electrode at different discharge/charge stages. **(c)** schematic illustration of reversible potassiation/depotassiation of the Se/N-3DMpC electrode

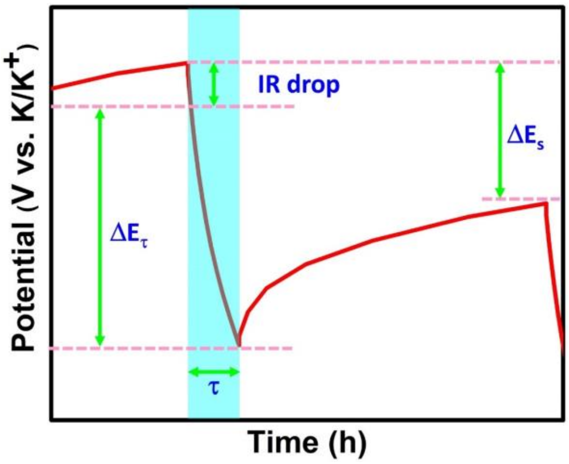

Fig. S29 The Dk value can be calculated by the simplified Fick's second law with the following equation:

$$D=\frac{4}{\pi\tau}\left(\frac{m_B V_M}{M_B A}\right)^2\left(\frac{\Delta E_s}{\Delta E_\tau}\right)^2$$

where M_B and V_M is the molar mass and molar volume of electrode material, m_B is the mass of electrode active material, τ is the pulse duration and A is the active surface area. ΔE_τ and ΔE_s are gained from GITT curves.

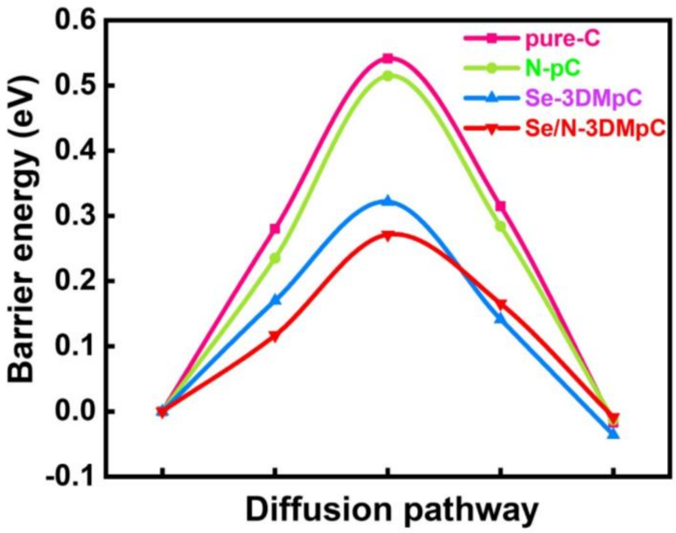

Fig. S30 K^+ diffusion energy barriers of the Se/N-3DMpC, Se-3DMpC, N-pC and pure-C

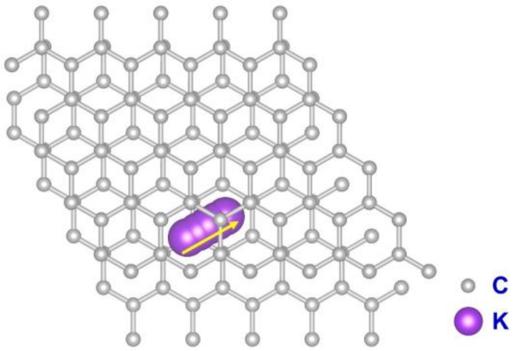

Fig. S31 Illustration of the K^+ diffusion path from on hollow position to the nearest hollow position in pure-C

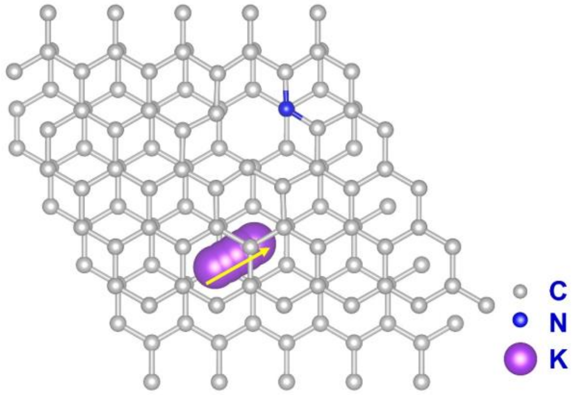

Fig. S32 Illustration of the K^+ diffusion path from on hollow position to the nearest hollow position in N-pC

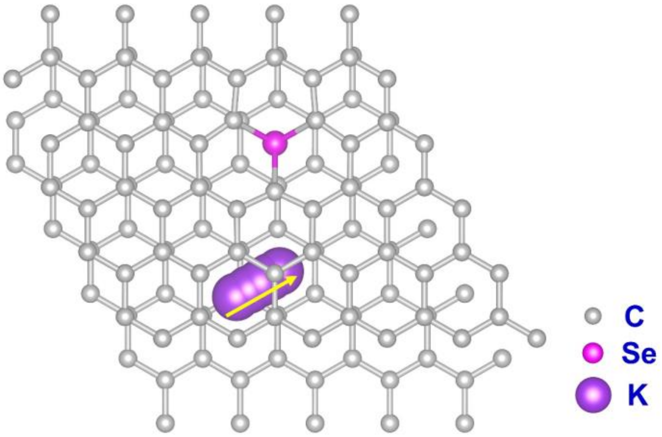

Fig. S33 Illustration of the K^+ diffusion path from on hollow position to the nearest hollow position in Se-3DMpC

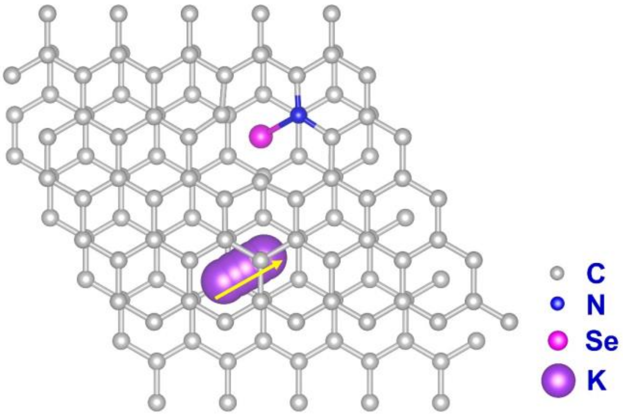

Fig. S34 Illustration of the K^+ diffusion path from on hollow position to the nearest hollow position in Se/N-3DMpC

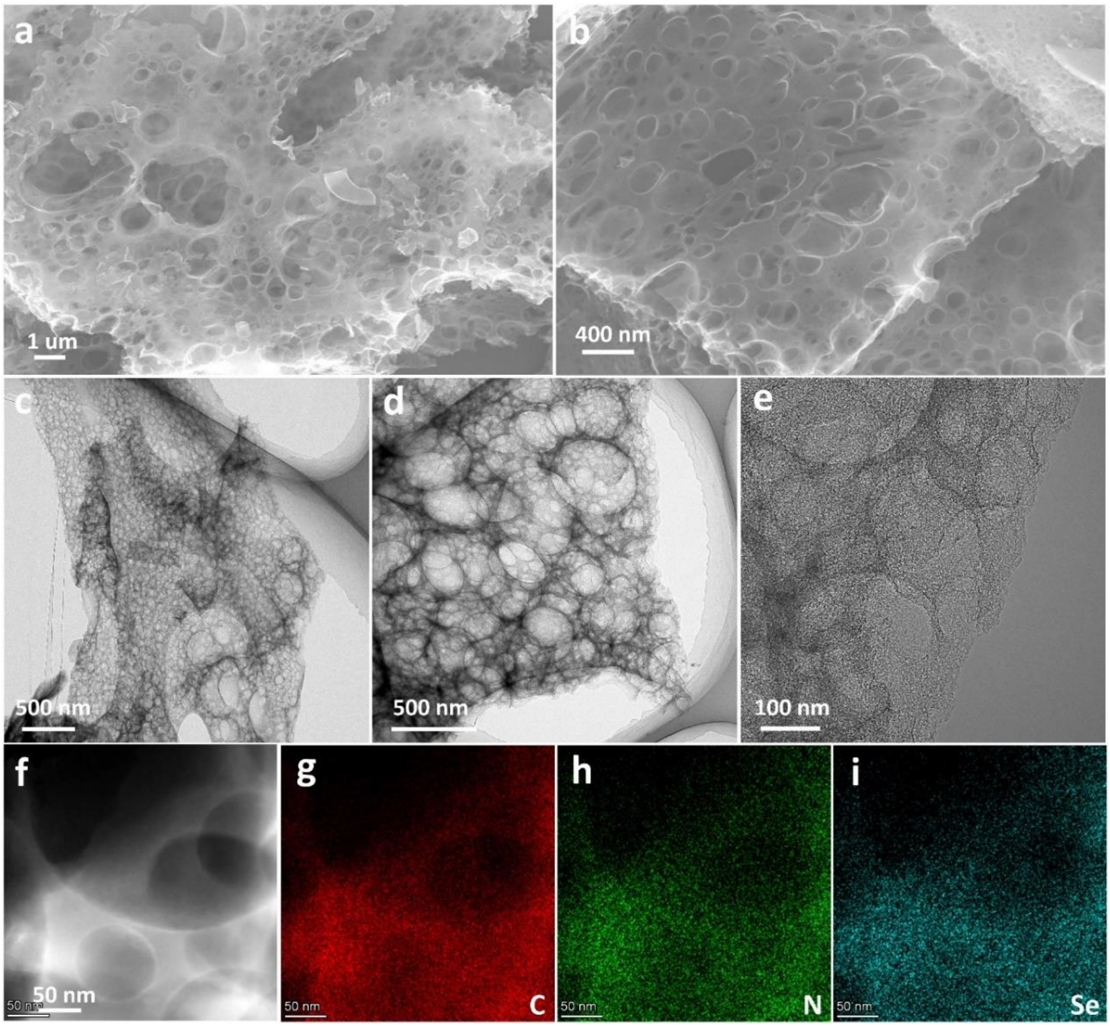

Fig. S35 (a, b) FESEM images, **(c-e)** TEM images and **(f-i)** HAADF-STEM and corresponding element mapping of the A-Se/N-3DMpC

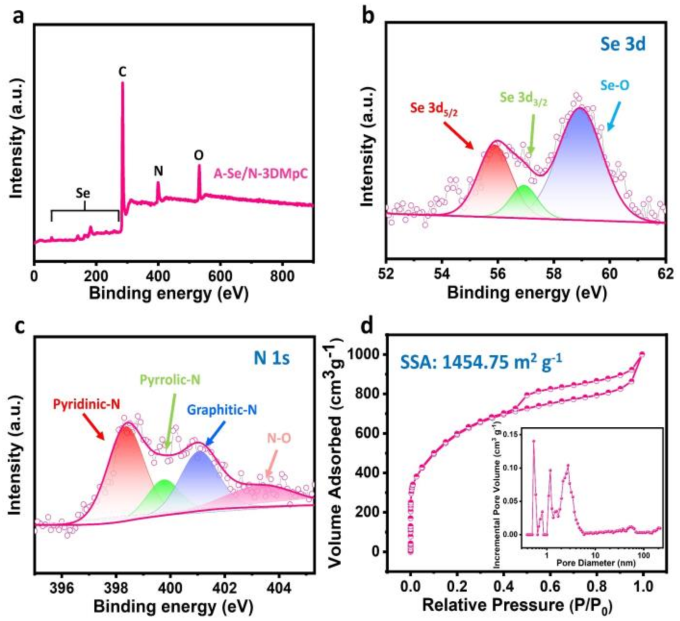

Fig. S36 (a) XPS survey spectra, **(b)** high-resolution Se 3d, **(c)** N 1s and **(d)** nitrogen adsorption-desorption isotherm curves (inset: pore size distribution) of the A-Se/N-3DMpC

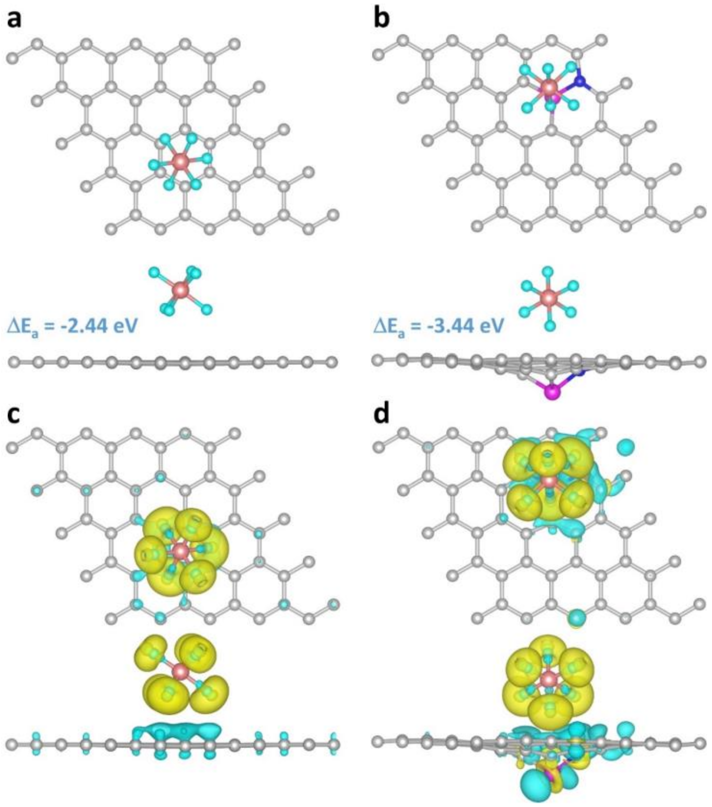

Fig. S37 Top and side view of the PF_6^- adsorbed on **(a)** pure carbon and **(b)** A-Se/N-3DMpC and the corresponding ΔE_a. Top and side view of the calculated charge density difference of PF_6^- adsorbed on **(c)** pure carbon and **(d)** A-Se/N-3DMpC. More electron transfer is observed in Se/N co-doped carbon, indicating stronger adsorption between the substrate and PF_6^-

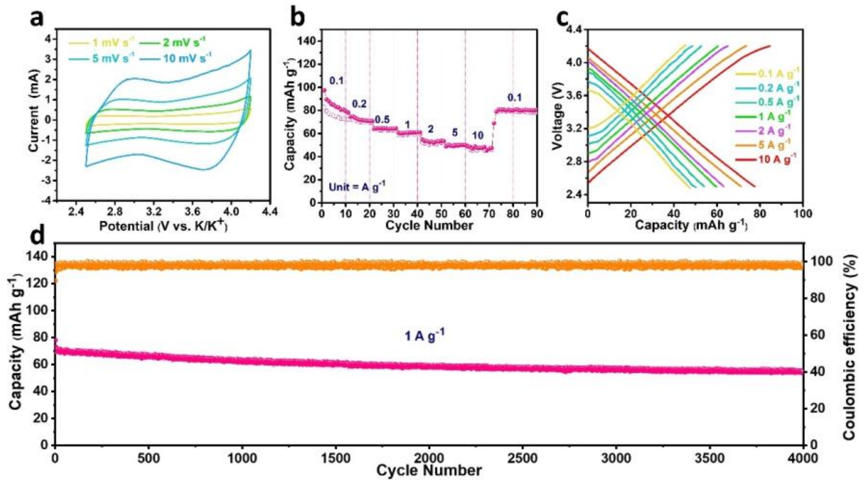

Fig. S38 (a) CV profiles at various sweep rates, **(b)** rate property, **(c)** charge/discharge curves at diversity current density and **(d)** long-term cycle performance at 1 A g^{-1} of the A-Se/N-3DMpC

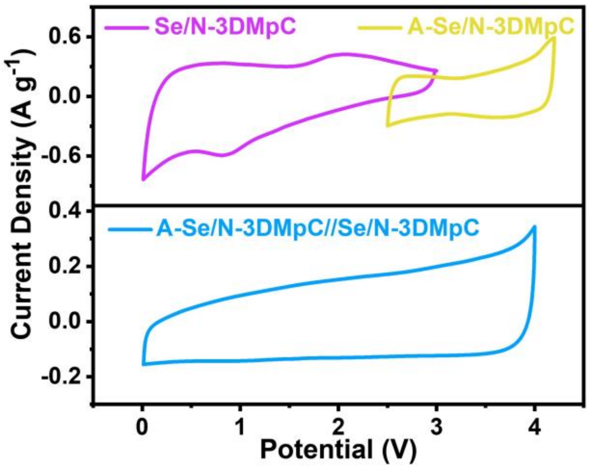

Fig. S39 CV curves of the Se/N-3DMpC and the A-Se/N-3DMpC electrodes in half cells (top) and full cell of PIHCs (bottom) at 1 mV s^{-1}

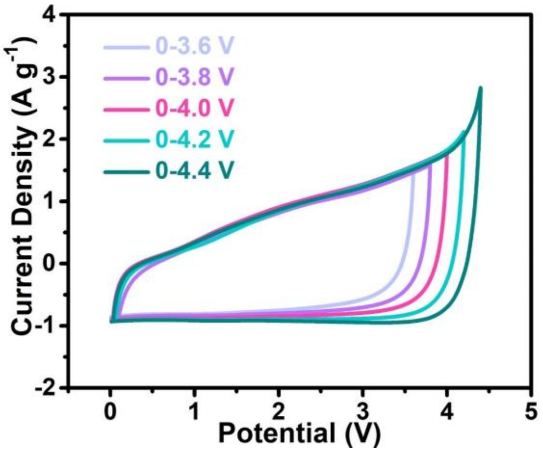

Fig. S40 CV curves of the A-Se/N-3DMpC//Se/N-3DMpC PIHCs device in various potential windows at a sweep rate of 2 mV s^{-1}

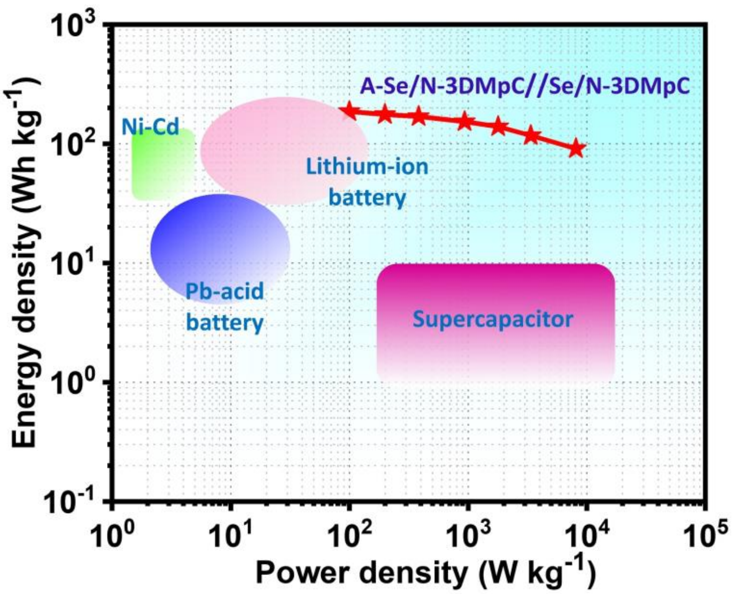

Fig. S41 Ragone plots of the A-Se/N-3DMpC//Se/N-3DMpC PIHCs device as compared with commercial devices for energy storage

Table S1 Comparison of the ICE value between Se/N-3DMpC electrode and previously reported carbon-based materials for PIBs

Materials	Current density (A g^{-1})	Initial coulombic efficiency	Refs.
Se/N-3DMpC	0.2	54.3%	This work
S/N@C	0.05	24.6%	[S1]
NOHC-1000	0.1	40.8%	[S2]
p-HNCs	0.1	37.9%	[S3]
HCNT	0.1	15%	[S4]
N/O Dual-Doped Hard Carbon	0.05	25%	[S5]
Sulfur-grafted hollow carbon spheres	0.025	51.4%	[S6]
Nitrogen-doped bamboo-like carbon	0.5	23.3%	[S7]
NHC_2-NH_3/Ar	0.1	15.8%	[S8]
N-doped hierarchical porous carbon	0.028	37.1%	[S9]
N-doped carbon fibers-650	0.025	49%	[S10]
3D nitrogen-doped framework carbon	0.1	24.3%	[S11]
carbon nanocage	0.279	40%	[S12]
nitrogen-doped carbon nanosheets	0.05	20%	[S13]
nitrogen-doped porous carbon	0.1	43.1%	[S14]

Table S2 Comparison of the cycling performance between Se-N-HPC electrode and previously reported carbon-based materials for PIBs

Materials	Voltage range(V)	Current density (mA g^{-1})	Cycle number	Specific capacity (mAh g^{-1})	Refs.
Se-N-HPC	0.01-3.0	200<br>2000	200<br>5000	335<br>214	This work
S/N@C	0.01-2.0	2000	900	65	[S1]
N/O dual-doped hard carbon	0.01-3.0	1000	5000	189.5	[S2]
Carbon quantum dot micelles tailored hollow carbon	0.01-3.0	1000	800	160	[S3]
Hierarchical carbon nanotube	0.01-2.5	100	500	210	[S4]
N/O-codoped hierarchical porous hard carbon	0.01-3.0	50	100	230.6	[S5]
N-doped bamboo-like carbon nanotubes	0.01-3.0	500	1000	204	[S7]
ultra-high pyrrolic/pyridinic-N-doped necklace-like hollow carbon film	0.01-2.6	1000	1600	161.3	[S8]
N-doped carbon nanofibers	0.01-3.0	2000	4000	146	[S10]
Graphitic carbon nanocage	0.01-2.5	56	100	195	[S12]
Nitrogen doped carbon nanosheets	0.01-3.0	1000	1000	151	[S13]
CFM-S30NG	0.01-2.5	1000	2000	188.8	[S15]
N/O co-doped mesoporous carbon octahedrons	0.01-3.0	1000	1300	100	[S16]
Carbon nanofiber foam	0.01-2.8	1000	2000	158	[S17]
S/O-codoped porous hard carbon microspheres	0.01-2.5	1000	2000	108.4	[S18]
Hollow interconnected neuron-like carbon architecture	0.01-2.0	280	500	150	[S19]

Defect-rich graphitic nanocarbons	0.01-2.0	200	200	189	[S20]
Amorphous ordered mesoporous carbon	0.01-2.6	1000	1000	146.5	[S21]
N/O codoped carbon hollow multihole bowls	0.01-2.5	1000	1000	133	[S22]
Pyridinic N-doped porous carbon monolith	0.01-3.0	1000	3000	152	[S23]
Graphdiyne-800	0.01-3.0	1000	2000	165	[S24]

Table S3 Comparison of the cycling stability of the state-of-the-art PIHCs with our work

PIHCs	Number of cycles	Capacity retention	Refs.
A-Se/N-3DMpC//Se/N-3DMpC	5000	83.2%	This work
NHCS//ANHCS	5000	80.4%	[S9]
CNS//FCDAC	5000	80%	[S25]
AC//$Ca_{0.5}Ti_2(PO_4)_3$@C	4000	75.9%	[S26]
AC//U-Co_2P@rGO-14	1000	68%	[S27]
$RuCo_2O_4$//AC	2500	82%	[S28]
SHNPC//AC	3750	75.4%	[S29]
$K_2Ti_6O_{13}$//NGC	5000	75.5%	[S30]
OLC//AC	6000	83%	[S31]
N-$MoSe_2$/G//AC	3000	75.2%	[S32]
PNC-MeNT//AC	3000	85.8%	[S33]
PNTCDA@900//AC	800	71.2%	[S34]
NCP//AC	5000	80.4%	[S35]
CBC@G//ACBC	5000	81.5%	[S36]
S-KTO@C//AC	3000	80.3%	[S37]
ALD KTO//AC	4000	70%	[S38]

Supplementary References

[S1] A. Mahmood, S. Li, Z. Ali, H. Tabassum, B. Zhu et al., Ultrafast sodium/potassium-ion intercalation into hierarchically porous thin carbon shells. Adv. Mater. **31**(2), e1805430 (2019). https://doi.org/10.1002/adma.201805430

[S2] R.C. Cui, B. Xu, H.J. Dong, C.C. Yang, Q. Jiang, N/O dual-doped environment-friendly hard carbon as advanced anode for potassium-ion batteries. Adv. Sci. **7**(5), 1902547 (2020). https://doi.org/10.1002/advs.201902547

[S3] W. Hong, Y. Zhang, L. Yang, Y. Tian, P. Ge et al., Carbon quantum dot micelles tailored hollow carbon anode for fast potassium and sodium storage. Nano Energy **65**, 104038 (2019). https://doi.org/10.1016/j.nanoen.2019.104038

[S4] Y. Wang, Z. Wang, Y. Chen, H. Zhang, M. Yousaf et al., Hyperporous sponge interconnected by hierarchical carbon nanotubes as a high-performance potassium-ion battery anode. Adv. Mater. **30**(32), e1802074 (2018). https://doi.org/10.1002/adma.201802074

[S5] J. Yang, Z. Ju, Y. Jiang, Z. Xing, B. Xi et al., Enhanced capacity and rate capability of nitrogen/oxygen dual-doped hard carbon in capacitive potassium-ion storage. Adv. Mater. **30**(4), 1700104 (2018). https://doi.org/10.1002/adma.201700104

[S6] J. Ding, H. Zhang, H. Zhou, J. Feng, X. Zheng et al., Sulfur-grafted hollow carbon spheres for potassium-ion battery anodes. Adv. Mater. **31**(30), e1900429 (2019). https://doi.org/10.1002/adma.201900429

[S7] Y. Liu, C. Yang, Q. Pan, Y. Li, G. Wang et al., Nitrogen-doped bamboo-like carbon nanotubes as anode material for high performance potassium ion batteries. J. Mater. Chem. A **6**(31), 15162-15169 (2018). https://doi.org/10.1039/c8ta04694h

[S8] W. Yang, J. Zhou, S. Wang, W. Zhang, Z. Wang et al., Freestanding film made by necklace-like n-doped hollow carbon with hierarchical pores for high-performance potassium-ion storage. Energy Environ. Sci. **12**(5), 1605-1612 (2019). https://doi.org/10.1039/c9ee00536f

[S9] D. Qiu, J. Guan, M. Li, C. Kang, J. Wei et al., Kinetics enhanced nitrogen-doped hierarchical porous hollow carbon spheres boosting advanced potassium-ion hybrid capacitors. Adv. Funct. Mater. **29**(32), 1903496 (2019). https://doi.org/10.1002/adfm.201903496

[S10] Y. Xu, C. Zhang, M. Zhou, Q. Fu, C. Zhao et al., Highly nitrogen doped carbon nanofibers with superior rate capability and cyclability for potassium ion batteries. Nat. Commun. **9**(1), 1720 (2018). https://doi.org/10.1038/s41467-018-04190-z

[S11] B. Yang, J. Chen, L. Liu, P. Ma, B. Liu et al., 3D nitrogen-doped framework carbon for high-performance potassium ion hybrid capacitor. Energy Storage Mater. **23**, 522-529 (2019). https://doi.org/10.1016/j.ensm.2019.04.008

[S12] B. Cao, Q. Zhang, H. Liu, B. Xu, S. Zhang et al., Graphitic carbon nanocage as a stable and high power anode for potassium-ion batteries. Adv. Energy Mater. **8**(25), 1801149 (2018). https://doi.org/10.1002/aenm.201801149

[S13] L. Liu, Y. Chen, Y. Xie, P. Tao, Q. Li et al., Understanding of the ultrastable k-ion storage of carbonaceous anode. Adv. Funct. Mater. **28**(29), 1801989 (2018). https://doi.org/10.1002/adfm.201801989

[S14] D. Li, X. Ren, Q. Ai, Q. Sun, L. Zhu et al., Facile fabrication of nitrogen-doped porous carbon as superior anode material for potassium-ion batteries. Adv. Energy Mater. **8**(34), 1802386 (2018). https://doi.org/10.1002/aenm.201802386

[S15] W. Yang, J. Zhou, S. Wang, Z. Wang, F. Lv et al., A three-dimensional carbon framework constructed by N/S co-doped graphene nanosheets with expanded interlayer spacing facilitates potassium ion storage. ACS Energy Lett. **5**(5), 1653-1661 (2020). https://doi.org/10.1021/acsenergylett.0c00413

[S16] G. Xia, C. Wang, P. Jiang, J. Lu, J. Diao et al., Nitrogen/oxygen co-doped mesoporous carbon octahedrons for high-performance potassium-ion batteries. J. Mater. Chem. A **7**(19), 12317-12324 (2019). https://doi.org/10.1039/c8ta12504j

[S17] H. Li, Z. Cheng, Q. Zhang, A. Natan, Y. Yang et al., Bacterial-derived, compressible, and hierarchical porous carbon for high-performance potassium-ion batteries. Nano Lett. **18**(11), 7407-7413 (2018). https://doi.org/10.1021/acs.nanolett.8b03845

[S18] M. Chen, W. Wang, X. Liang, S. Gong, J. Liu et al., Sulfur/oxygen codoped porous hard carbon microspheres for high-performance potassium-ion batteries. Adv. Energy Mater. 8(19), 1800171 (2018). https://doi.org/10.1002/aenm.201800171

[S19] D.S. Bin, X.J. Lin, Y.G. Sun, Y.S. Xu, K. Zhang et al., Engineering hollow carbon architecture for high-performance k-ion battery anode. J. Am. Chem. Soc. **140**(23), 7127-7134 (2018). https://doi.org/10.1021/jacs.8b02178

[S20] W. Zhang, J. Ming, W. Zhao, X. Dong, M.N. Hedhili et al., Graphitic nanocarbon with engineered defects for high-performance potassium-ion battery anodes. Adv. Funct. Mater. **29**(35), 1903641 (2019). https://doi.org/10.1002/adfm.201903641

[S21] W. Wang, J. Zhou, Z. Wang, L. Zhao, P. Li et al., Short-range order in mesoporous carbon boosts potassium-ion battery performance. Adv. Energy Mater. **8**(5), 1701648 (2018). https://doi.org/10.1002/aenm.201701648

[S22] Z. Zhang, B. Jia, L. Liu, Y. Zhao, H. Wu et al., Hollow multihole carbon bowls: A stress-release structure design for high-stability and high-volumetric-capacity potassium-ion batteries. ACS Nano **13**(10), 11363-11371 (2019). https://doi.org/10.1021/acsnano.9b04728

[S23] Y. Xie, Y. Chen, L. Liu, P. Tao, M. Fan et al., Ultra-high pyridinic N-doped porous carbon monolith enabling high-capacity K-ion battery anodes for both half-cell and full-cell applications. Adv. Mater. **29**(35), 1702268 (2017). https://doi.org/10.1002/adma.201702268

[S24] Y. Yi, J. Li, W. Zhao, Z. Zeng, C. Lu et al., Temperature-mediated engineering of graphdiyne framework enabling high-performance potassium storage. Adv. Funct. Mater. **30**(31), 2003039 (2020). https://doi.org/10.1002/adfm.202003039

[S25] J. Chen, B. Yang, H. Hou, H. Li, L. Liu et al., Disordered, large interlayer spacing, and oxygen-rich carbon nanosheets for potassium ion hybrid capacitor. Adv. Energy Mater. **9**(19), 1803894 (2019). https://doi.org/10.1002/aenm.201803894

[S26] Z. Zhang, M. Li, Y. Gao, Z. Wei, M. Zhang et al., Fast potassium storage in hierarchical $Ca_{0.5}Ti_2(Po_4)_3$@c microspheres enabling high-performance potassium-ion capacitors. Adv. Funct. Mater. **28**(36), (2018). https://doi.org/10.1002/adfm.201802684

[S27] Y. Wang, Z. Zhang, G. Wang, X. Yang, Y. Sui et al., Ultrafine Co_2P nanorods wrapped by graphene enable a long cycle life performance for a hybrid potassium-ion capacitor. Nanoscale Horiz. **4**(6), 1394-1401 (2019). https://doi.org/10.1039/c9nh00211a

[S28] D.P. Dubal, N.R. Chodankar, R. Holze, D.H. Kim, P. Gomez-Romero, Ultrathin mesoporous $RuCo_2O_4$ nanoflakes: An advanced electrode for high-performance asymmetric supercapacitors. ChemSusChem **10**(8), 1771-1782 (2017). https://doi.org/10.1002/cssc.201700001

[S29] H. Luo, M. Chen, J. Cao, M. Zhang, S. Tan et al., Cocoon silk-derived, hierarchically porous carbon as anode for highly robust potassium-ion hybrid capacitors. Nano-Micro Lett. **12**(1), (2020). https://doi.org/10.1007/s40820-020-00454-w

[S30] S. Dong, Z. Li, Z. Xing, X. Wu, X. Ji et al., Novel potassium-ion hybrid capacitor based on an anode of $K_2Ti_6O_{13}$ microscaffolds. ACS Appl. Mater. Inter. **10**(18), 15542-15547 (2018). https://doi.org/10.1021/acsami.7b15314

[S31] J. Chen, B. Yang, H. Li, P. Ma, J. Lang et al., Candle soot: Onion-like carbon, an advanced anode material for a potassium-ion hybrid capacitor. J. Mater. Chem. A. **7**(15), 9247-9252 (2019). https://doi.org/10.1039/c9ta01653h

[S32] Y. Yi, Z. Sun, C. Li, Z. Tian, C. Lu et al., Designing 3d biomorphic nitrogen-doped $MoSe_2$/graphene composites toward high-performance potassium-ion capacitors. Adv. Funct. Mater. **30**(4), (2019). https://doi.org/10.1002/adfm.201903878

[S33] J. Li, L. Yu, Y. Li, G. Wang, L. Zhao et al., Phosphorus-doping-induced kinetics modulation for nitrogen-doped carbon mesoporous nanotubes as

superior alkali metal anode beyond lithium for high-energy potassium-ion hybrid capacitors. Nanoscale **13**(2), 692-699 (2021). https://doi.org/10.1039/d0nr06888h

[S34] Y. Liu, Q. Ru, Y. Gao, Q. An, F. Chen et al., Constructing volcanic-like mesoporous hard carbon with fast electrochemical kinetics for potassium-ion batteries and hybrid capacitors. Appl. Surf. Sci. 525 (2020). https://doi.org/10.1016/j.apsusc.2020.146563

[S35] M. Liu, L. Chang, J. Wang, J. Li, J. Jiang et al., Hierarchical n-doped carbon nanosheets submicrospheres enable superior electrochemical properties for potassium ion capacitors. J. Power Sources 469 (2020). https://doi.org/10.1016/j.jpowsour.2020.228415

[S36] D. Qiu, J. Guan, M. Li, C. Kang, J. Wei et al., Cucurbit[6]uril-derived nitrogen-doped hierarchical porous carbon confined in graphene network for potassium-ion hybrid capacitors. Adv. Sci. **7**(20), 2001681 (2020). https://doi.org/10.1002/advs.202001681

[S37] S. Zhao, L. Dong, B. Sun, K. Yan, J. Zhang et al., $K_2Ti_2O_5$ @C microspheres with enhanced K^+ intercalation pseudocapacitance ensuring fast potassium storage and long-term cycling stability. Small **16**(4), e1906131 (2020). https://doi.org/10.1002/smll.201906131

[S38] D. Li, C. Liu, D. Huang, M. Zhang, X. Zhang et al., Atomic layer deposition regulating hydrated $K_2Ti_6O_{13}$ nanobelts on graphene platform with accelerated solid solution potassiation for potassium ion capacitors. Chem. Eng. J. (2020). https://doi.org/10.1016/j.cej.2020.128048
